# Single-cell RNA sequencing reveals sex differences in the subcellular composition and associated gene-regulatory network activity of human carotid plaques

**DOI:** 10.1038/s44161-025-00628-y

**Published:** 2025-04-10

**Authors:** Katyayani Sukhavasi, Giuseppe Mocci, Lijiang Ma, Chani J. Hodonsky, Ernest Diez Benevante, Lars Muhl, Jianping Liu, Sonja Gustafsson, Byambajav Buyandelger, Simon Koplev, Urban Lendahl, Michael Vanlandewijck, Prosanta Singha, Tiit Örd, Mustafa Beter, Ilakya Selvarajan, Johanna P. Laakkonen, Marika Väli, Hester M. den Ruijter, Mete Civelek, Ke Hao, Arno Ruusalepp, Christer Betsholtz, Heli Järve, Jason C. Kovacic, Clint L. Miller, Casey Romanoski, Minna U. Kaikkonen, Johan L. M. Björkegren

**Affiliations:** 1 Department of Cardiac Surgery and The Heart Clinic, Tartu University Hospital and Department of Cardiology, Institute of Clinical Medicine, Tartu University, Tartu, Estonia; 2 Department of Medicine, Karolinska Institutet, Karolinska Universitetssjukhuset, Huddinge, Sweden; 3 Department of Genetics and Genomic Sciences, Institute of Genomics and Multiscale Biology, Icahn School of Medicine at Mount Sinai, New York, NY USA; 4 Robert M. Berne Cardiovascular Research Center, University of Virginia, Charlottesville, VA USA; 5 Department of Genome Sciences, University of Virginia, Charlottesville, VA USA; 6 Laboratory of Experimental Cardiology, Department of Cardiology, Division Heart and Lungs, University Medical Center Utrecht, Utrecht University, Utrecht, The Netherlands; 7 Cancer Research UK Cambridge Institute, University of Cambridge, Cambridge, UK; 8 Department of Cell and Molecular Biology, Karolinska Institutet, Stockholm, Sweden; 9 Department of Immunology, Genetics, and Pathology, Rudbeck Laboratory, Uppsala University, Uppsala, Sweden; 10 A. I. Virtanen Institute for Molecular Sciences, University of Eastern Finland, Kuopio, Finland; 11 Department of Pathological Anatomy and Forensic Sciences, Tartu University, Tartu, Estonia; 12 Department of Biomedical Engineering, University of Virginia, Charlottesville, VA USA; 13 Department of Vascular Surgery and The Surgery Clinic, Tartu University Hospital, Tartu, Estonia; 14 Cardiovascular Research Institute, Icahn School of Medicine at Mount Sinai, New York, NY USA; 15 Victor Chang Cardiac Research Institute, Darlinghurst, New South Wales Australia; 16 St. Vincent’s Clinical School, University of NSW, Sydney, New South Wales Australia; 17 Department of Public Health Sciences, University of Virginia, Charlottesville, VA USA; 18 Department of Cellular and Molecular Medicine, College of Medicine, University of Arizona, Tucson, AZ USA; 19 Clinical Gene Networks AB, Stockholm, Sweden

**Keywords:** Regulatory networks, Cardiovascular diseases

## Abstract

Carotid stenosis causes ischemic stroke in both sexes, but the clinical presentation and plaque characteristics differ. Here we run deep single-cell sequencing of 7,690 human carotid plaque cells from male and female patients. While we found no sex differences in major cell types, we identified a predominance of the osteogenic phenotype in smooth muscle cells, immunomodulating macrophages (MPs) and endothelial cells (ECs) undergoing endothelial-to-mesenchymal transition in females. In males, we found smooth muscle cells with the chondrocytic phenotype, MPs involved in tissue remodeling and ECs with angiogenic activity. Sex-biased subcellular clusters were integrated with tissue-specific gene-regulatory networks (GRNs) from the Stockholm–Tartu Atherosclerosis Reverse Network Engineering Task study. We identified GRN195 involved in angiogenesis and T cell-mediated cytotoxicity in male ECs, while in females, we found GRN33 and GRN122 related to TREM2^−^/TREM1^+^ MPs and endothelial-to-mesenchymal transition. The impact of GRN195 on EC proliferation in males was functionally validated, providing evidence for potential therapy targets for atherosclerosis that are sex specific.

## Abstract (web summary)

Using deep sequencing of human carotid plaque cells from male and female patients with carotid stenosis, Sukhavasi et al. identify sex-biased cell-type-specific gene-regulatory networks and different phenotypes in smooth muscle cells, including an osteogenic phenotype in females and a chondrocytic phenotype in males.

## Main

Coronary and carotid atherosclerosis are late-onset diseases and the main underlying causes of myocardial infarction and stroke in both males and females^1^. In females, however, the development of carotid stenosis is associated with a disproportionate risk of stroke, mortality and disability^2,3^. This is probably explained by differences observed in the clinical manifestation and histological appearance of carotid stenosis between the sexes (that is, plaque size, composition and morphology)^4,5^. In males, the clinical manifestation of carotid stenosis typically occurs earlier in life, more frequently presenting with carotid plaques characterized by a lipid-rich necrotic core and intraplaque hemorrhage^6^. In females, however, probably due to their premenopausal period with atherosclerosis-protective sex hormones, the clinical manifestation of carotid stenosis occurs later in life and then more frequently with a plaque erosion phenotype linked to a higher risk of thrombus formation^7^.

The main cell types of atherosclerotic plaques are endothelial cells (ECs), smooth muscle cells (SMCs) and different forms of CD45^+^ immune and inflammatory cells. However, in recent single-cell RNA sequencing (scRNA-seq) studies, it has become clear that these major cell types undergo transformation into subtypes paralleled with the progression of atherosclerosis^8^. Arterial wall SMCs have been shown to transform into osteogenic-like cells during late-stage atherosclerosis, both in mice^9^ and humans^10^. SMCs seem, however, to have a multipotent differentiation capacity including generation of pro-atherogenic macrophage (MP)-like^11^ and fibrochondrocyte-like cells, or, alternatively, reversal back to a more native athero-protective contractile form of SMCs^12^. Besides SMCs, the single-cell atherosclerosis landscape of CD45^+^ immune cells and in part ECs have been studied both in humans^13^ and mice^9,11,14,15^. In mice, resident-like MPs have been found in both healthy and atherosclerotic aortas. In contrast, in atherosclerotic aortas, mainly two populations of MPs were detectable, comprising lipid-poor pro-inflammatory MPs^16^ and lipid-laden triggering receptor expressed on myeloid cells 2 (TREM2)-expressing MPs^17^, the latter potentially with a less inflammatory phenotype^11^. Lineage tracing studies in mice have revealed that a portion of lesion foam cells derived from SMCs^10,11^ may transform further to become more atherogenic in the form of osteogenic-like cells^9^. Overall, these observations in mice have been supported by the comprehensive studies of CD45^+^ cells in human carotid plaques, which further provided evidence of the importance of endothelial–mesenchymal transitions (endoMT) and a decline in CD4^+^ and CD8^+^ cell cytotoxicity^4^. The latter contrasted an earlier study characterizing symptomatic carotid plaques in which a distinct subset of activated and differentiated CD4^+^ T cells was described^13^. Although we have provided initial support for sex-biased arterial wall networks identified in bulk-RNA data^18,19^, scRNA-seq studies specifically addressing possible sex differences in the subcellular composition of carotid plaques that may underlie the sex-diverse clinical manifestations and histological appearance of carotid stenosis are lacking.

Herein, by deep sequencing fluorescence activated cell sorting (FACS)-sorted cells isolated from carotid plaques obtained during endarterectomy in women and men, we provide strong support for marked differences in the subcellular composition of carotid plaques in relation to sex. Next, to provide clinical and pathophysiological context, 15 sex-biased subcellular clusters were integrated with 135 tissue-specific gene-regulatory networks (GRNs) inferred from two arterial wall and four metabolic tissues of the Stockholm–Tartu Atherosclerosis Reverse Network Engineering Task (STARNET) study^5,18^. Through this process, the cellular origin, reproducibility and clinical implications of the female GRN33 and GRN122 involving TREM2^−^/TREM1^+^high MPs and endoMT, respectively, and the male GRN195 of the vasa vasorum ECs involving angiogenesis and T cell-mediated cytotoxicity were identified and subsequently validated in six independent arterial wall and cell-type-specific RNA-seq datasets. In addition, by overexpressing its top key drivers, plasmalemma vesicle-associated protein (*PLVAP*) and family with sequence similarity 110 member D (*FAM110D*), the presence and overall function of GRN195 were successfully evaluated in cultured human aortic ECs (HAECs).

## Results

### Characteristics of patients and major carotid plaque cell types

**Fig. 1:**
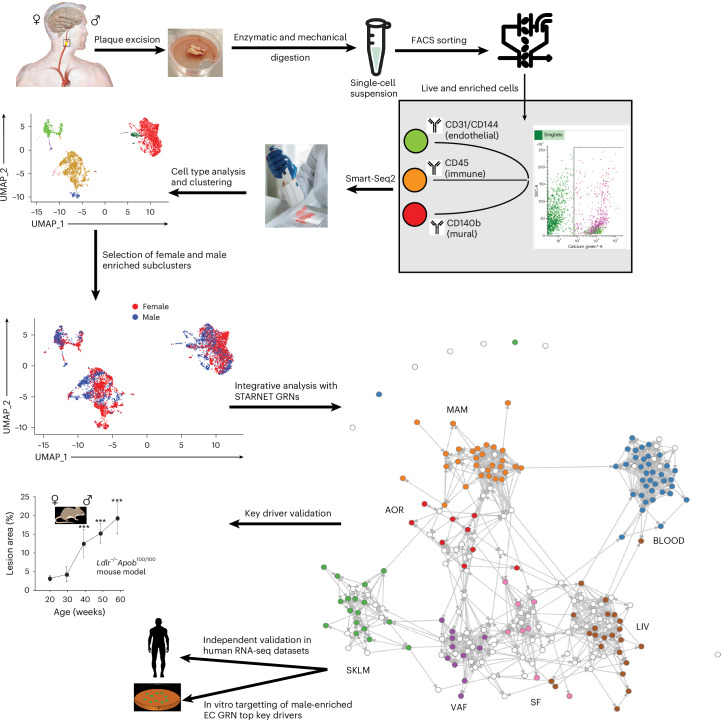
Schematic overview of the overall study design. A schematic illustrating the key stages and components of the overall study design. Credit: human with carotid artery, ref. ^70^ under a Creative Commons license CC BY 4.0; Eppendorf tube, TogoTV (© 2016 DBCLS TogoTV) under a Creative Commons license CC BY 4.0; cytometry, Flaticon.com; PCR plate, Pexels.com; mouse and Petri dish, Pixabary.com; mouse model, ref. ^45^ under a Creative Commons license CC BY 4.0; GRN network, ref. ^5^, Springer Nature America. LIV, liver.

**Extended Data Fig. 1:**
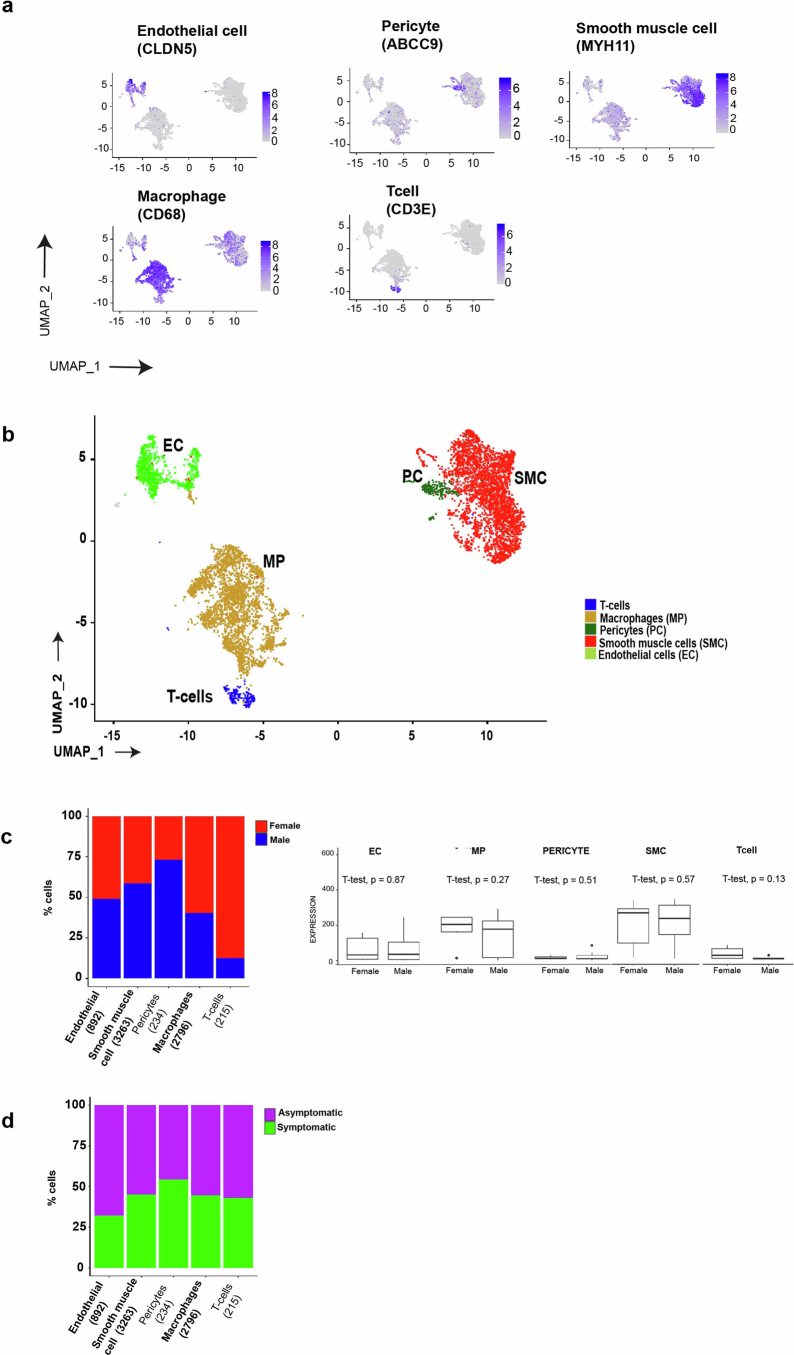
Major cell types of the human carotid plaque. **a**) Uniform Manifold Approximation and Projection **(**UMAP) featuring single cell gene expression levels of established cell-type markers of endothelial cells (ECs, Claudin5 (CLDN5)), macrophages (MPs, (CD68)), pericytes (PCs, ATP binding cassette subfamily C member 9 (ABCC9)), smooth muscle cells (SMCs, Myosin11 (MYH11)) and T-cells (T-cell surface glycoprotein CD3 Epsilon chain (CD3E)). **b**) UMAP of major cell types. **c**) Bar plots showing the relative contribution of major cell types isolated from female (n = 7) and male (n = 8) carotid plaques. (), indicates the total number of cells in each cluster. In the boxplot, centre line denotes the median value (50th percentile), the box contains the 25th to 75th percentiles of the dataset. *P*-values were calculated using students´ T-test (two-tailed) by comparing female and male major cell type numbers. No adjustment was made. **d**) Bar plots showing the relative contributions of major cell types isolated from asymptomatic (n = 7) or symptomatic (n = 8) carotid plaques. (), indicates the total number of cells in each cluster. Source data

Other than an expected higher median age of the female compared with the male patients with carotid stenosis, the basic clinical characteristics of the 15 study patients were comparable across sexes (Supplementary Tables 1 and 2). To study the subcellular nature of the major cell types in carotid plaques isolated from these patients, Smart-Seq2 (ref. ^19^) was performed on a total of 7,690 cells isolated by FACS (PDGFRb^+^, CD31^+^, CD144^+^ and CD45^+^) (Supplementary Fig. 1), whereof 7 patients were female (*n* = 3,841 cells) and 8 patients were male (*n* = 3,849 cells) (Fig. 1). According to established cell-type markers (Extended Data Fig. 1a), initial clustering^20^ of the scRNA-seq data revealed three major cell types corresponding to ECs, MPs and SMCs, as well as two smaller cell-type clusters represented by T cells and pericytes (Extended Data Fig. 1b and Supplementary Tables 3–11). Within the three major cell types, the numbers of single cells isolated from each of the sexes were similar (Extended Data Fig. 1c). In contrast, subcellular clusters of these major cell types (Supplementary Tables 12–33) were markedly sex biased representing a diversity of pathobiological functions according to gene ontology (GO) (Supplementary Tables 34–55).

### Sex differences of the carotid plaque SMC subcellular clusters

**Fig. 2:**
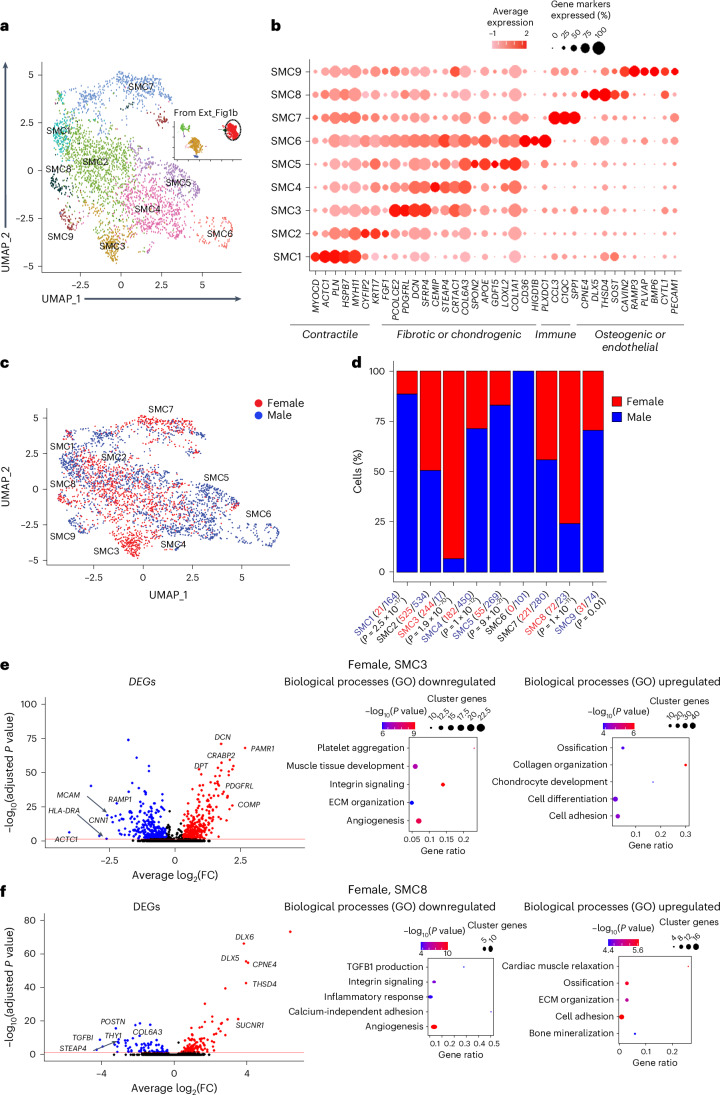
Single-cell RNA sequence characterization of SMC subcellular clusters in carotid plaques of female and male patients. **a**, Subcellular clusters of human SMC carotid plaques visualized with UMAP. Ext_Fig1b, UMAP of major cell types. This shows that SMC cell types circled in black have been subclustered to get 9 subcellular clusters, SMC1–SMC9. **b**, Dot plot showing expression levels of functional gene markers within the SMC subclusters. SMC6 was excluded from further analysis because it emerged from only one patient. **c**, UMAP of SMC subcellular clusters highlighted for cells isolated from carotid plaques of female and male patients. **d**, Bar plots showing the relative sex specificity of SMC subcluster cells. The total number of cells in each category are shown in parentheses. *P* values were calculated using chi-square statistics assessing the statistical significance between observed and expected values. **e**, Volcano plot showing differentially expressed SMC3 genes (red, upregulated; blue, downregulated) using all other SMC subclusters as background. Cell-type-specific gene enrichment was calculated using the Wilcoxon rank-sum test (log_2_(fold) > 0.3, Bonferroni-adjusted *P* < 0.005). Dot plots show top-ranked biological processes according to GO. Gene ratios (*x* axis) are the relative number of subcluster genes in relation to the total gene count in each GO category. Dot size indicates the actual number of subcluster genes in each GO category. GO enrichment −log_10_(*P* values) were calculated with Fisher exact test. Cluster genes indicate the number of SMC3 subcluster genes overlapping with the GO category. ECM, extracellular matrix. **f**, Volcano plot showing differentially expressed SMC8 genes (red, upregulated; blue, downregulated) using all other SMC subclusters as background. Cell-type-specific gene enrichment was calculated using Wilcoxon rank–sum test (log_2_(fold) > 0.3, Bonferroni-adjusted *P* < 0.005). Dot plots show top-ranked biological processes according to GO. Gene ratios (*x* axis) are the relative number of subcluster genes in relation to the total gene count in each GO category. Dot size indicates the actual number of subcluster genes in each GO category. GO enrichment −log_10_(*P* values) were calculated with Fisher exact test. Cluster genes indicate the number of SMC8 subcluster genes overlapping with the GO category. Source data

Subclustering of 3,263 carotid plaque SMCs resulted in 9 SMC subtypes (Fig. 2a–c; SMC1–9) represented by 1,351 female and 1,912 male SMCs. Across sexes within a trajectory from SMC1 to SMC9 (Fig. 2b), the expression levels of SMC markers for contractile functions (actin alpha cardiac muscle 1, *ACTC1*; Myosin11, *MYH11*) gradually decreased whereas markers for phenotypic switching (cartilage acidic protein 1, *CRTAC1*; sclerostin, *SOST*; complement component 1qC, *C1QC*) gradually increased, consistent with the transformation of contractile SMCs into immune-activated, chondrogenic and osteogenic phenotypes, as previously reported^21^.

Within this transformation, the SMC subclusters showed noticeable sex biases: 93% (*n* = 244/261) and 76% (*n* = 72/95) of the cells in SMC3 and SMC8, respectively, were isolated from female patients (Fig. 2c,d). To explore the overall functions of these clusters, their contents of established phenotypic markers (Methods) and the GO annotations of all genes in each cluster were examined. In SMC3, markers of a myofibroblast-like phenotype were identified (Fig. 2e, left panel) consistent with its top-ranked biological process, collagen fibril organization (GO:0030199) and ossification (GO:0001503) (Fig. 2e, right panel). In SMC8, osteogenic-like phenotype markers dominated (Fig. 2f, left panel) consistent with its top-ranked biological process bone mineralization (GO:0030500) (Fig. 2f, right panel).

**Extended Data Fig. 2:**
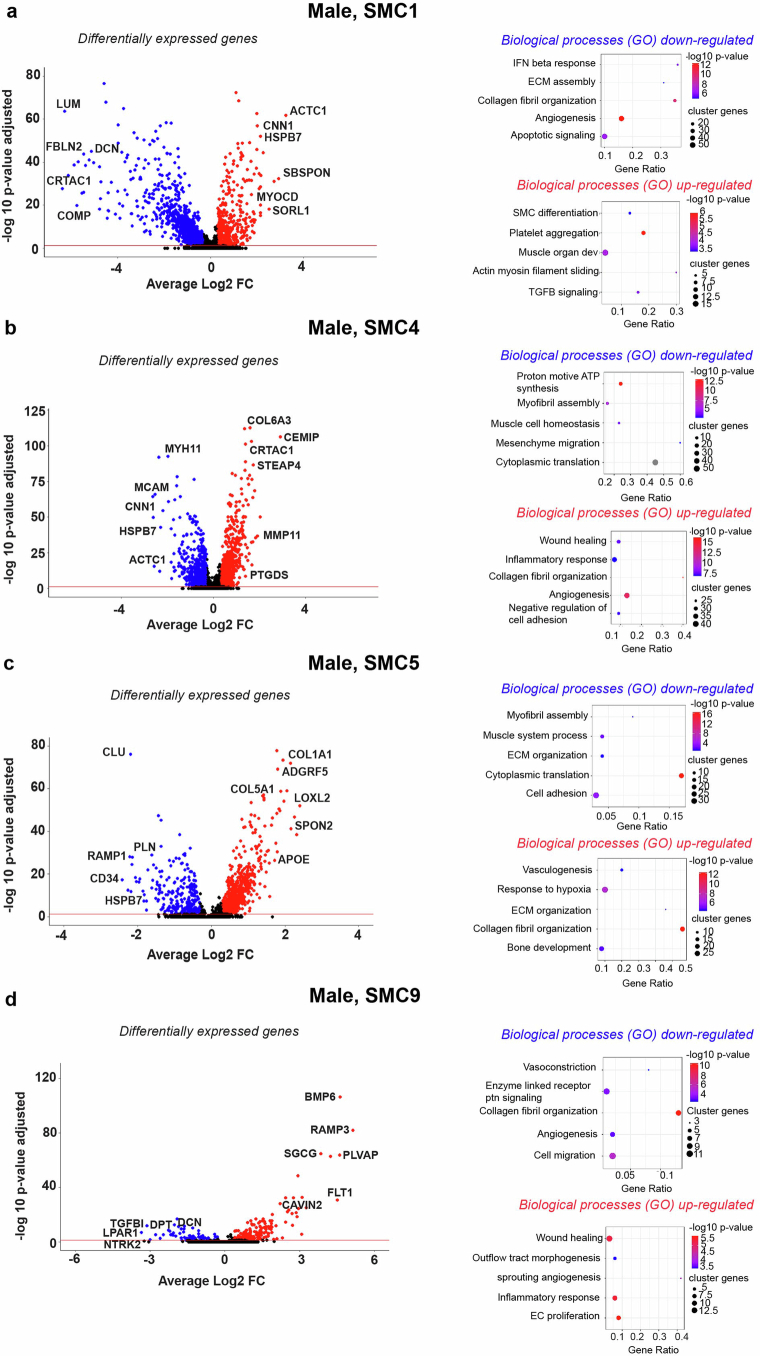
Carotid plaque SMC subcellular genes and related biological processes. **a**-**d**) Volcano plots (left) showing SMC1 (**a**), SMC4 (**b**), SMC5 (**c**), and SMC9 (**d**) subcluster DEGs using all other SMC subclusters as background. Cell type–specific gene enrichment was calculated using Wilcoxon Rank-Sum test (log_2_-fold >0.3, Bonferroni-adjusted *P* < 0.005). Dot plots show top-ranked biological processes according to GO. *Gene Ratios* (x-axis) are the relative number of subcluster genes in relation to the total gene count in each GO category. Dot size indicates the actual number of subcluster genes in each GO category. GO enrichment −log10 *P* values were calculated with Fisher exact test. Cluster genes indicate the number of subcluster genes overlapping with the GO category. Source data

Male-dominated SMC subclusters were SMC1 (*n* = 165/185), SMC4 (*n* = 450/632), SMC5 (*n* = 269/324) and SMC9 (*n* = 74/105) (Fig. 2d). In the male SMC1 subcluster, markers associated with smooth muscle contraction were identified (Extended Data Fig. 2a, left panel) consistent with its top-ranked biological process muscle organ development (GO:0007517) (Extended Data Fig. 2a, bottom right panel). In SMC4 and SMC5, markers of a chondrocyte-like phenotype prevailed (SMC4, Extended Data Fig. 2b, left panel and SMC5, Extended Data Fig. 2c, left panel), which corresponded well with their top-ranked biological processes: collagen fibril organization GO:0030199) and chondrocyte development (GO:0002063) (Extended Data Fig. 2b,c, bottom right panel). The male SMC9 subcluster was characterized by endothelial-like phenotype markers (Extended Data Fig. 2d, left panel) corresponding to its top-ranked biological processes of positive regulation of endothelial cell proliferation (GO:0001938) and angiogenesis (GO:0045766) (Extended Data Fig. 2d, bottom right panel). The 100% male SMC6 (*n* = 101 cells) originated from a single patient and was therefore excluded from further analysis.

### Sex differences of the carotid plaque MP subcellular clusters

**Fig. 3:**
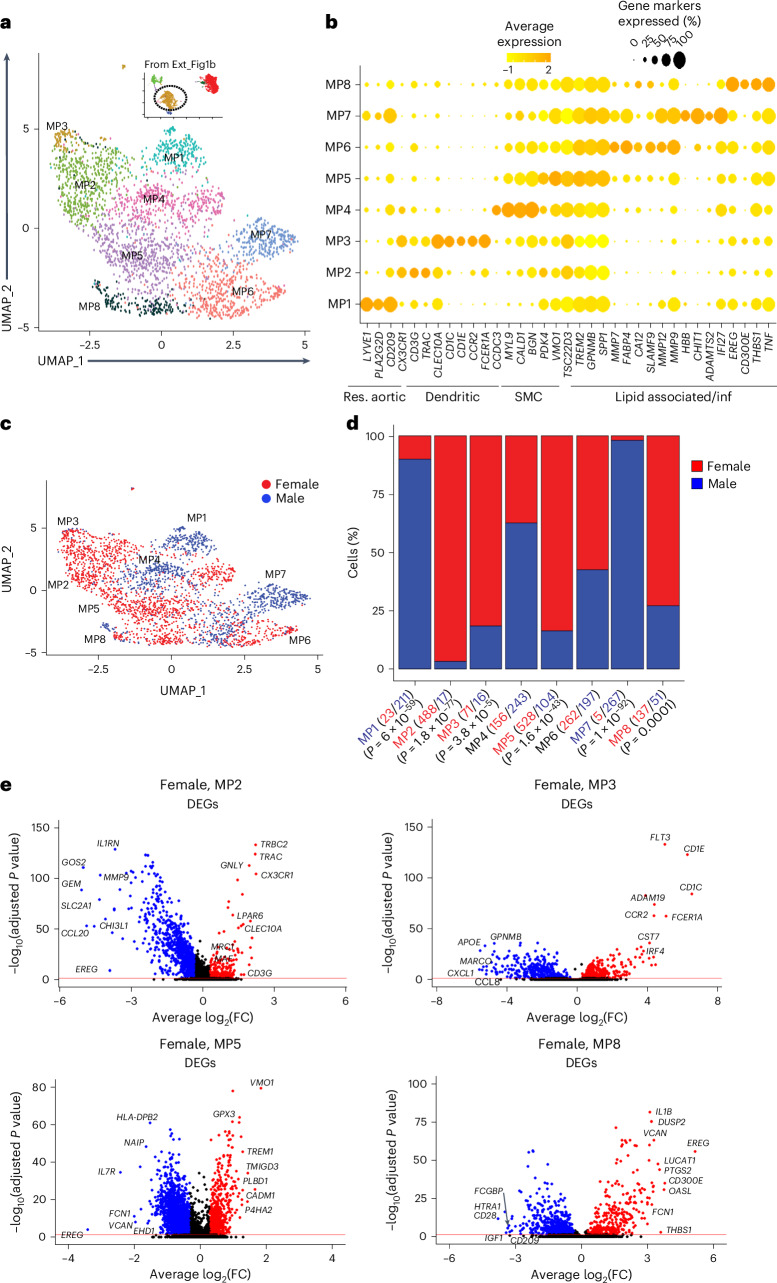
Single-cell RNA sequence characterization of MP subcellular clusters in carotid plaques of female and male patients. **a**, Subcellular clusters of human carotid plaque MPs visualized with UMAP. **b**, Dot plot showing expression levels of functional gene markers within the MP subclusters. Res., resident; inf, inflammatory. **c**, UMAP of MP subcellular clusters highlighted for cells isolated from carotid plaques of female and male patients. **d**, Bar plots showing the relative sex specificity of MP subcluster cells. The total number of cells in each category are shown in parentheses. *P* values were calculated using chi-square statistics assessing the statistical significance between observed and expected values. **e**, Volcano plots showing DEGs in the indicated MP subclusters using all other MP subclusters as background (red, upregulated; blue, downregulated). Cell-type-specific gene enrichment was calculated using Wilcoxon rank-sum test (log_2_(fold) > 0.3, Bonferroni-adjusted *P* < 0.005). Source data

Subclustering of 2,796 carotid plaque MPs resulted in 8 subclusters (Fig. 3a–c; MP1–8). Across sex in a trajectory from MP1 to MP8, markers for resident-like (lymphatic vessel endothelial hyaluronan receptor 1, *LYVE1*; *DC-SIGN*, *CD209*), dendritic-like (C-type lectin domain family 10 member A, *CLEC10A*; cluster of differentiation 1, *CD1C*), smooth muscle cell-like (myosin light chain 9, *MYL9*; biglycan, *BGN*) and lipid-associated and inflammatory-like (triggering receptor expressed on myeloid cells 2, *TREM2*; matrix metallopeptidase 9, *MMP9*) evolved (Fig. 3b). Of the 2,769 MPs, 1,670 and 1,126 were female and male MPs, respectively (Fig. 3c).

**Extended Data Fig. 3:**
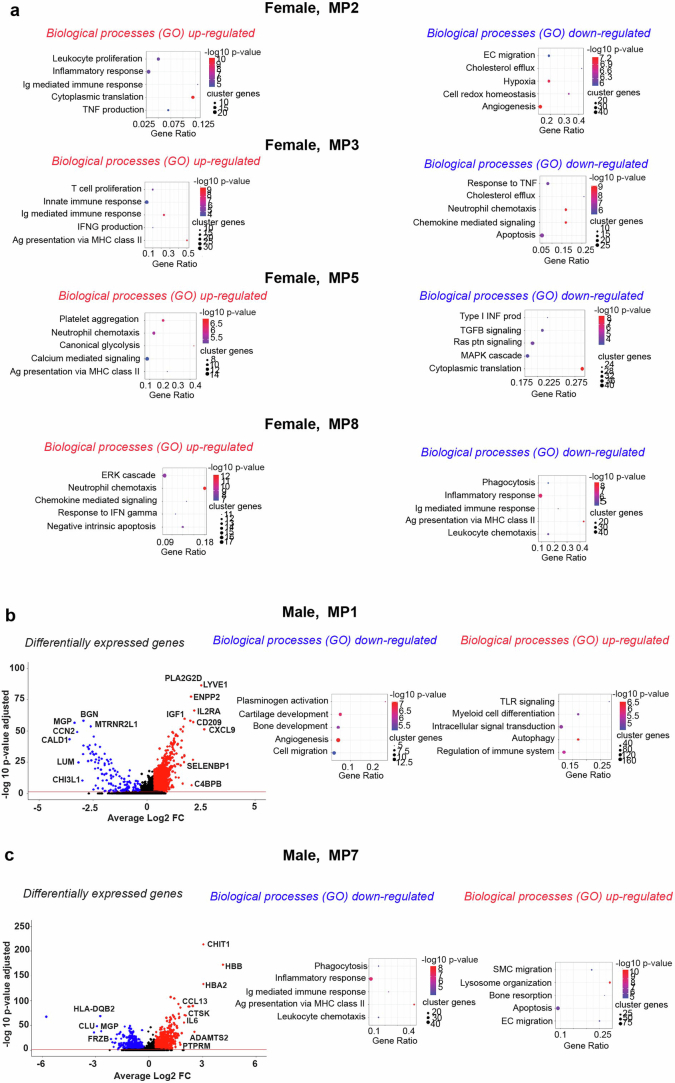
Carotid plaque MP subcellular genes and related biological processes. **a**) Dot plots of MP2, MP3, MP5 and MP8 show top-ranked biological processes according to GO. *Gene Ratios* (x-axis) are the relative number of subcluster genes in relation to the total gene count in each GO category. Dot size indicates the actual number of subcluster genes in each GO category. GO enrichment −log10 *P* values were calculated with Fisher exact test. Cluster genes indicate the number of subcluster genes overlapping with the GO category. **b**) Volcano plots (left) of MP1 subcluster DEGs using all other MP subclusters used as background. Cell type–specific gene enrichment was calculated using Wilcoxon Rank-Sum test (log_2_-fold >0.3, Bonferroni-adjusted *P* < 0.005). Dot plots (right) show top-ranked biological processes according to GO. *Gene Ratios* (x-axis) are the relative number of subcluster genes in relation to the total gene count in each GO category. Dot size indicates the actual number of subcluster genes in each GO category. GO enrichment −log10 *P* values were calculated with Fisher exact test. Cluster genes indicate the number of subcluster genes overlapping with the GO category. **c**) Volcano plots (left) of MP7 subcluster DEGs using all other MP subclusters used as background. Cell type–specific gene enrichment was calculated using Wilcoxon Rank-Sum test (log_2_-fold >0.3, Bonferroni-adjusted *P* < 0.005). Dot plots (right) show top-ranked biological processes according to GO. *Gene Ratios* (x-axis) are the relative number of subcluster genes in relation to the total gene count in each GO category. Dot size indicates the actual number of subcluster genes in each GO category. GO enrichment −log10 *P* values were calculated with Fisher exact test. Cluster genes indicate the number of subcluster genes overlapping with the GO category. Source data

Among these MP subclusters, MP2 (*n* = 488 out of 505), MP3 (*n* = 71 out of 87), MP5 (*n* = 528 out of 632) and MP8 (*n* = 137 out of 188) were female dominant (Fig. 3d). Overall, the combined biological processes of these female subclusters were largely immune response related including antigen presentation via MHC class II (GO:0019886), immunoglobulin-mediated immune response (GO:0002381) and cellular response to interferon-gamma (GO:0071346) (Fig. 3e and Extended Data Fig. 3a).

Male-dominant MP subclusters were MP1 (*n* = 211 out of 234) and MP7 (*n* = 267 out of 272) (Fig. 3d). In the MP1 subcluster, markers associated with a resident-like MP phenotype were identified (Extended Data Fig. 3b, left panel), which largely corresponded to its top-ranked biological processes regulation of autophagy (GO:0010506) and toll-like receptor signaling pathway (GO:0002224) (Extended Data Fig. 3b, right panel). In MP7, markers associated with hemoglobin and tissue remodeling (Extended Data Fig. 3c, left panel) corresponded well with its top-ranked biological processes: lysosome organization (GO:0007040), positive regulation of blood vessel endothelial cell migration (GO:0043536) and regulation of bone resorption (GO:0045124) (Extended Data Fig. 3c, right panel).

### Sex differences of the carotid plaque EC subcellular clusters

**Fig. 4:**
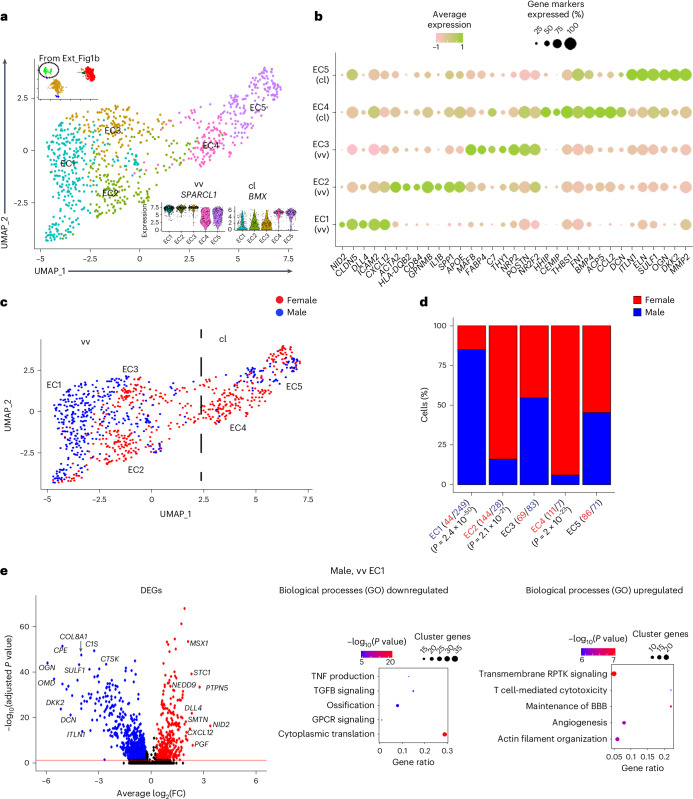
Single-cell RNA sequence characterization of EC subcellular clusters in carotid plaques of female and male patients. **a**, Subcellular clusters of human carotid plaque ECs visualized with UMAP. vv, vasa vasorum; cl, carotid lumen. The inserted violin plots show the EC subcluster expression levels of the capillary EC marker, *SPARCL1*, and the luminal EC marker, *VWF*. **b**, Dot plot showing the expression levels of functional gene markers within the five EC subclusters. **c**, UMAPs of EC subcellular clusters highlighted for cells isolated from carotid plaques of female and male patients. **d**, Bar plots showing the relative sex specificity of cells in each EC subcluster. The total number of cells collected in each category are shown in parentheses. *P* values were calculated using chi-square statistics assessing the statistical significance between observed and expected values. **e**, Volcano plots showing differentially expressed EC1 genes (red, upregulated; blue, downregulated) using all other EC subcluster as background. Cell-type-specific gene enrichment was calculated using Wilcoxon rank-sum test (log_2_(fold) > 0.3, Bonferroni-adjusted *P* < 0.005). Dot plots show top-ranked biological processes according to GO. Gene ratios (*x* axis) are the relative number of subcluster genes in relation to the total gene count in each GO category. Dot size indicates the actual number of subcluster genes in each GO category. GO enrichment −log_10_(*P* values) were calculated with Fisher exact test. Cluster genes indicate the number of EC1 subcluster genes overlapping with the GO category. GPCR, G protein-coupled receptor; RPTK, receptor protein tyrosine kinase; BBB, blood–brain barrier. Source data

Subclustering of 892 ECs from the carotid plaque resulted in 5 subclusters: EC1–EC3 from the vasa vasorum as indicated by their expression of *SPARCL1* and EC4 and EC5 from the carotid lumen as indicated by their expression of *BMX* (Fig. 4a–c; EC1–5). Among the vasa vasorum subclusters, EC1 was characterized by angiogenesis markers (Claudin5, *CLDN5*; Delta-like canonical Notch ligand 4, *DLL4*), EC2 by markers of SMC and immune cells (smooth muscle alpha-2 actin, *ACTA2*; major histocompatibility complex, class II, DQ beta 2, *HLA-DQB2*) and EC3 by markers of inflammation (nuclear receptor subfamily 2, group F, member 2, *NR2F2*; THYmocyte differentiation antigen 1, *THY1*) (Fig. 4b). The carotid lumen EC4 and EC5 were characterized by markers of endothelial progenitor cells (prominin1, *PROM1*; hedgehog-interacting protein, *HHIP*) and the extracellular matrix (elastin, ELN; osteoglycin, *OGN*), respectively (Fig. 4b).

**Extended Data Fig. 4:**
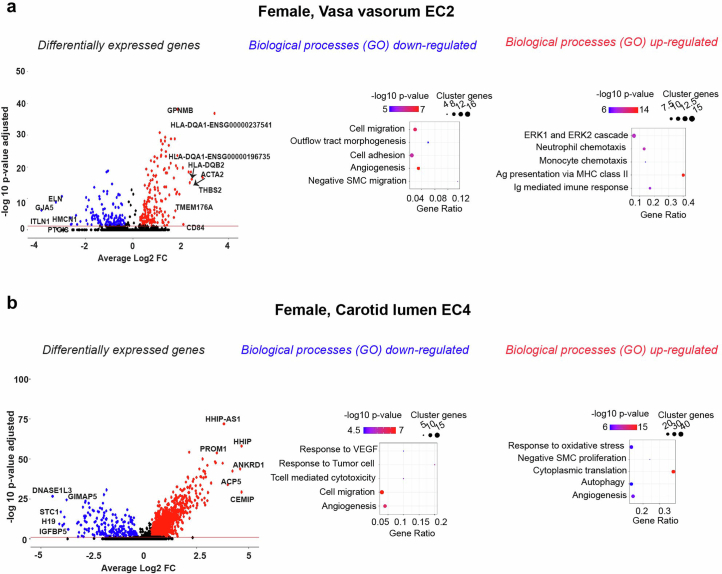
Carotid plaque EC subcellular genes and related biological processes. **a**) Volcano plots showing differentially expressed EC2 genes (red, up-regulated; blue, down-regulated) using all other EC subclusters as background. Cell type–specific gene enrichment was calculated using Wilcoxon Rank-Sum test (log_2_-fold >0.3, Bonferroni-adjusted *P* < 0.005). Dot plots show top-ranked biological processes according to GO. *Gene Ratios* (x-axis) are the relative number of subcluster genes in relation to the total gene count in each GO category. Dot size indicates the actual number of subcluster genes in each GO category. GO enrichment −log10 *P* values were calculated with Fisher exact test. Cluster genes indicate the number of EC2 subcluster genes overlapping with the GO category. **b**) Volcano plots showing differentially expressed EC4 genes (red, up-regulated; blue, down-regulated) using all other EC subclusters as background. Cell type–specific gene enrichment was calculated using Wilcoxon Rank-Sum test (log_2_-fold >0.3, Bonferroni-adjusted *P* < 0.005). Dot plots show top-ranked biological processes according to GO. *Gene Ratios* (x-axis) are the relative number of subcluster genes in relation to the total gene count in each GO category. Dot size indicates the actual number of subcluster genes in each GO category. GO enrichment −log10 *P* values were calculated with Fisher exact test. Cluster genes indicate the number of EC4 subcluster genes overlapping with the GO category. Source data

Of the 892 ECs, 454 and 438 were isolated from female and male carotid plaques, respectively (Fig. 4c). The vasa vasorum EC1 was dominated by ECs from male patients (*n* = 249 out of 293; Fig. 4d). In EC1, markers of EC proliferation and migration and *RAP1* signaling transduction dominated (Fig. 4e, left panel), which was paralleled by its top-ranked biological processes, positive regulation of angiogenesis (GO:0045766) and T cell-mediated cytotoxicity (GO:0001916) (Fig. 4e, right panel). Female-dominant EC subclusters were the vasa vasorum-derived EC2 (*n* = 144 out of 172) and the carotid-lumen-derived EC4 (*n* = 111 out of 118) (Fig. 4c,d). In EC2, SMC markers of endothelial-to-mesenchymal transformation (endMT) and acquisition of antigen-presenting cell functions suggested that ECs in this cluster also undergo endothelial-to-immune cell-like transformation (endICLT)^22^ (Extended Data Fig. 4a, left panel). The top-ranked biological processes of EC2 were MHC class II antigen presentation (GO:0019886) and immunoglobulin-mediated immune response (GO:0002381) (Extended Data Fig. 4a, right panel). In EC4, markers of tissue-resident vascular endothelial progenitors were identified suggesting a vascular repair phenotype (Extended Data Fig. 4b, left panel), which was corroborated by its top-ranked biological process response to oxidative stress (GO:0006979) and autophagy (GO:0006914) (Extended Data Fig. 4b, right panel).

### Integration of sex-biased SMC clusters with human GRNs

**Fig. 5:**
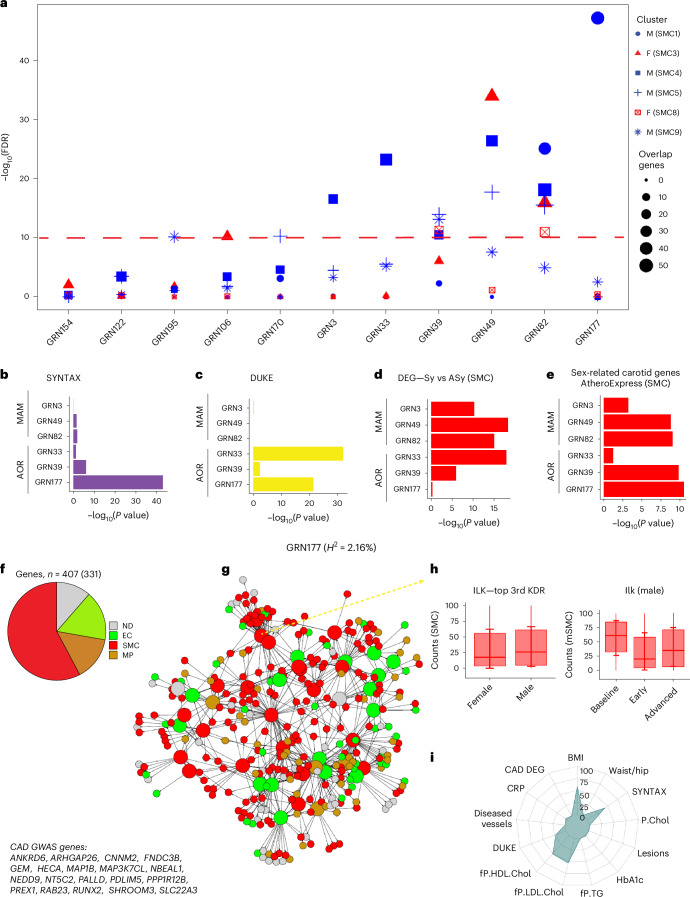
Enrichment of sex-diversified SMC subcluster genes in human GRNs. **a**, Dot plot showing 11 top-ranked arterial wall GRNs (*x* axis) according to their enrichments in genes of sex-specified SMC subclusters in the carotid plaques. The *y* axis shows −log_10_(10% FDR) (highlighted). Dot size indicates the number of genes overlapping between the SMC subclusters and GRNs. M, male; F, female. **b**, Horizontal bar graph showing the statistical enrichments (*x* axis, −log_10_(*P* value)) of genes associated with SYNTAX scores in the arterial wall-specific GRNs above −log(10% FDR) in **a**. **c**, Horizontal bar graph showing the statistical enrichments (*x* axis, −log_10_(*P* value)) of genes associated with Duke scores in the arterial-wall-specific GRNs above −log(10% FDR) in **a**. **d**, Horizontal bar graph showing the enrichment significances (*x* axis, −log_10_(*P* value)) of DEGs in indicated SMC subclusters between symptomatic (Sy) and asymptomatic (Asy) carotid plaques in the arterial wall GRNs above −log(10% FDR) in **a**. **e**, Horizontal bar graph showing the enrichment significances (*x* axis, −log_10_(*P* value)) in indicated SMC subclusters of DEGs in Athero-Express scRNA-seq carotid plaque data^55^ between 20 females and 26 males in the arterial wall GRNs above −log(10% FDR) in **a**. **f**, Pie chart showing the relative cell type specificity of genes in GRN177 according to the scRNA-seq data (Methods). Below the pie chart are abbreviations of GRN177 GWAS CAD candidate genes. **g**, GRN177 color coded according to the cell type specificity. Bigger-sized nodes are key driver genes. **h**, Box plot (left) showing sex-specific expression of top-ranked key driver genes isolated from female (*n* = 7) and male (*n* = 8) carotid plaques and (right) corresponding expression pattern during the progression of atherosclerosis in female (*n* = 18) or male (*n* = 28) *Ldlr*^−^^*/*−^*Apob*^100/100^ mice (Methods). ND, not determined; KDR: key driver; mSMC: mouse SMC clusters in *Ldlr*^−/−^*Apob*^100/100^ mice. Top or rank, the key driver’s hierarchical ranking in the GRN. *H*^2^, broad sense heritability contribution of GRN177 (%). The red center line denotes the median value (50th percentile), and the red box contains the 25th to 75th percentiles of the dataset. The red whiskers mark the 5th and 95th percentiles. **i**, Radar plot showing the statistical significance of key cardiometabolic phenotype associations with GRN177. The significance of GRN–phenotype associations (−log_10_; *P* = 0–100) was calculated by aggregating GRN gene-level phenotype associations (Pearson correlation two tailed *t*-test) corrected for the total number of STARNET GRNs (*n* = 135) and the number of genes in each GRN using the Benjamini–Hochberg procedure. fP.HDL.Chol, fasting plasma high-density lipoprotein cholesterol levels; fP.LDL.Chol, fasting plasma low-density lipoprotein cholesterol levels; fP.TG, fasting plasma triglyceride levels; HbA1c, hemoglobin A1C/glycated hemoglobin; P.Chol, plasma cholesterol levels. Source data

**Fig. 6:**
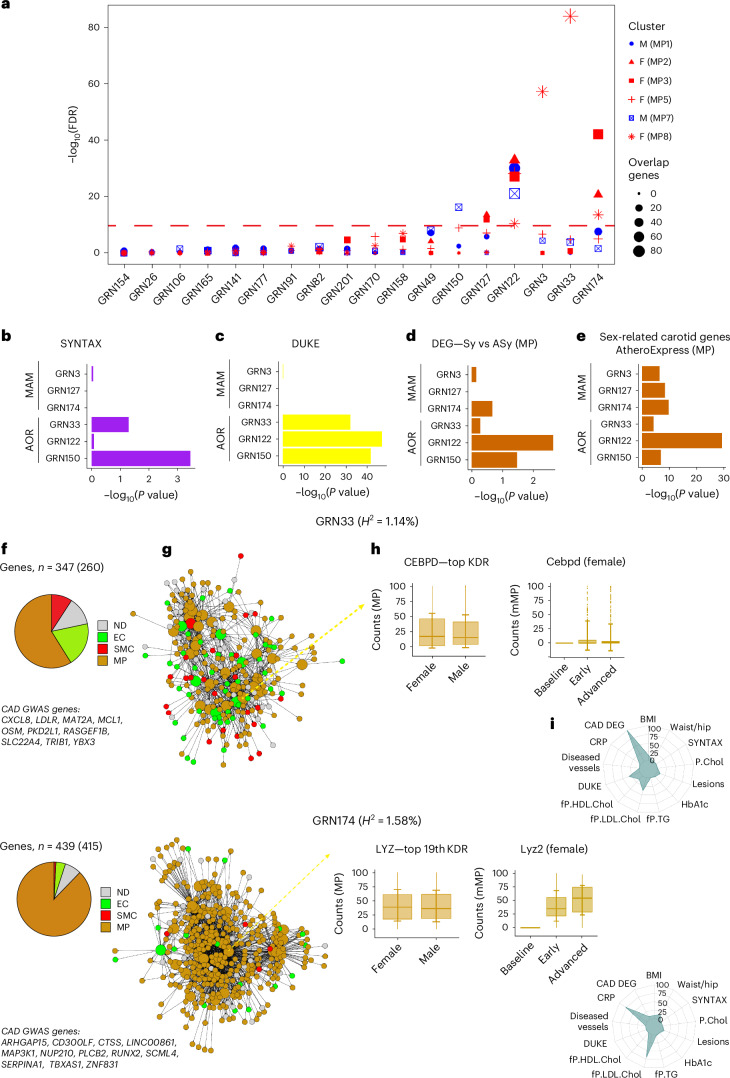
Enrichment of sex-diversified MP subcluster genes in human GRNs. **a**, Dot plot showing 18 top-ranked arterial wall GRNs (*x* axis) according to their enrichments in genes of sex-specified MP subclusters in the carotid plaques. The *y* axis shows −log_10_(10% FDR) (highlighted). Dot size indicates the number of genes overlapping between MP subclusters and GRNs. **b**, Horizontal bar graph showing the statistical enrichments (*x* axis, −log_10_(*P* value)) of genes associated with SYNTAX scores of the arterial-wall-specific GRNs above −log(10% FDR) in **a**. **c**, Horizontal bar graph showing the statistical enrichments (*x* axis, −log_10_(*P* value)) of genes associated with Duke scores in the arterial-wall-specific GRNs above −log(10% FDR) in **a**. **d**, Horizontal bar graph showing the enrichment significances (*x* axis, −log_10_(*P* value)) of DEGs in indicated MP subclusters between symptomatic and asymptomatic carotid plaques in the arterial wall GRNs above −log(10% FDR) in **a**. **e**, Horizontal bar graph showing the enrichment significances (*x* axis, −log_10_(*P* value)) in indicated MP subclusters of DEGs in Athero-Express scRNA-seq carotid plaque data^55^ between 20 females and 26 males in the arterial-wall GRNs above −log(10% FDR) in **a**. **f**, Pie chart showing the relative cell type specificity of genes in GRN33 (top) and GRN174 (bottom) according to the scRNA-seq data (Methods). Below the pie chart are abbreviations of GRN33 (top) and GRN174 (bottom) GWAS CAD candidate genes. **g**, GRN33 (top) and GRN174 (bottom) color coded according to cell type specificity. Bigger-sized nodes are the key driver genes. **h**, Box plots (left) showing sex-specific expression of top-ranked key drivers isolated from female (*n* = 7) and male (*n* = 8) carotid plaques and (right) corresponding expression patterns during the progression of atherosclerosis in female (*n* = 18) or male (*n* = 28) *Ldlr*^−*/*−^*Apob*^100/100^ mice (Methods). mMP, mouse MP clusters in *Ldlr*^−/−^*Apob*^100/100^ mice. Top or rank, the key driver’s hierarchical ranking in the GRN. *H*^2^, broad sense heritability contributions of GRN33 (top) and GRN174 (bottom) (%). The golden center line denotes the median value (50th percentile), and the golden box contains the 25th to 75th percentiles of the dataset. The golden whiskers mark the 5th and 95th percentiles. **i**, Radar plot showing the statistical significance of key cardiometabolic phenotype associations with GRN33 (top) and GRN174 (bottom). The significance of GRN–phenotype associations (−log_10_; *P* = 0–100) was calculated by aggregating GRN gene-level phenotype associations (Pearson correlation two-tailed *t*-test) corrected for the total number of STARNET GRNs (*n* = 135) and the number of genes in each GRN using the Benjamini–Hochberg procedure. Source data

**Fig. 7:**
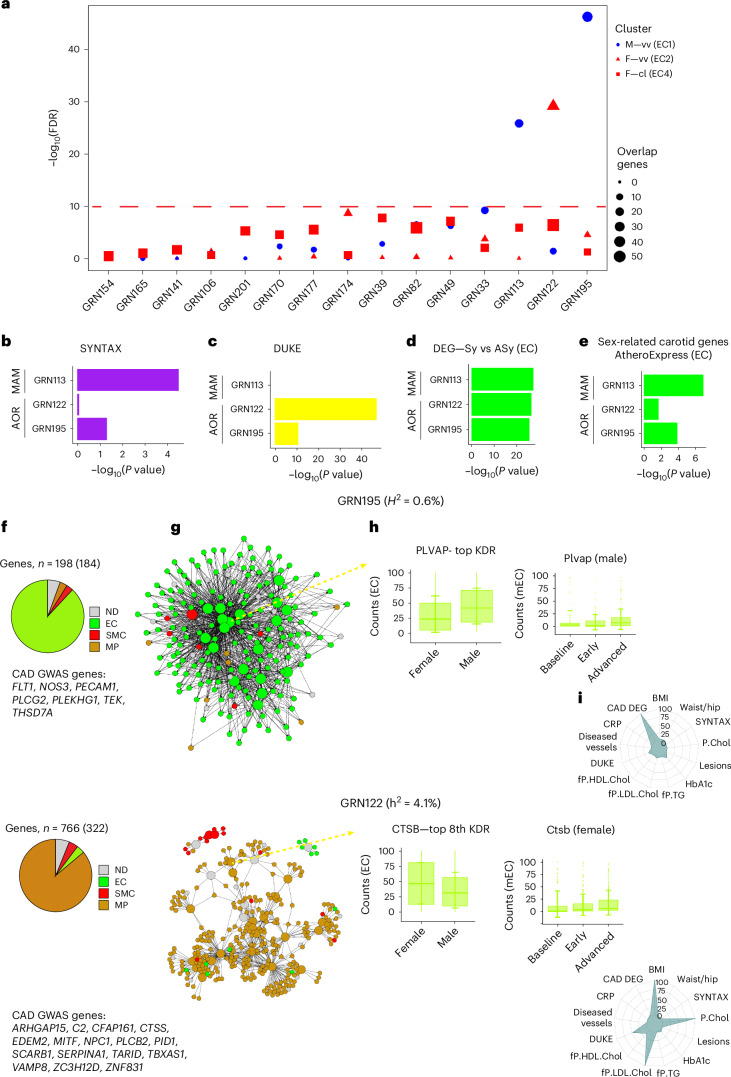
Enrichments of sex-diversified EC subcluster genes in human GRNs. **a**, Dot plot showing 15 top-ranked arterial wall GRNs (*x* axis) according to their enrichments in genes of sex-specified EC subclusters in the carotid plaques. The *y* axis shows −log(10% FDR) (highlighted). Dot size indicates the number of genes overlapping between EC subclusters and GRNs. **b**, Horizontal bar graph showing the statistical enrichments (*x* axis, −log_10_(*P* value)) of genes associated with SYNTAX scores of the arterial-wall-specific GRNs above −log(10% FDR) in **a**. **c**, Horizontal bar graph showing the statistical enrichments (*x* axis, −log_10_(*P* value)) of genes associated with Duke scores in the arterial-wall-specific GRNs above −log(10% FDR) in **a**. **d**, Horizontal bar graph showing the enrichment significances (*x* axis, −log_10_(*P* value)) of DEGs in indicated EC subclusters between symptomatic and asymptomatic carotid plaques in the arterial-wall GRNs above −log(10% FDR) in **a**. **e**, Horizontal bar graph showing the enrichment significances (*x* axis, −log_10_(*P* value)) in indicated EC subclusters of DEGs in Athero-Express scRNA-seq carotid plaque data^55^ between 20 females and 26 males in the arterial-wall GRNs above −log(10% FDR) in **a**. **f**, Pie chart showing the relative cell type specificity of genes in GRN195 (top) and GRN122 (bottom) according to the scRNA-seq data (Methods). Below the pie chart are abbreviations of GRN195 (top) and GRN122 (bottom) GWAS CAD candidate genes. **g**, GRN195 (top) and GRN122 (bottom) color coded according to the cell type specificity. Bigger-sized nodes are key driver genes. **h**, Bar plot (left) showing sex-specific expression of top-ranked key drivers isolated from female (*n* = 7) and male (*n* = 8) carotid plaques and (right) corresponding expression patterns during the progression of atherosclerosis in female (*n* = 18) or male (*n* = 28) *Ldlr*^*−/*^^*−*^*Apob*^100/100^ mice (Methods). Top or rank, key driver’s hierarchical ranking in the GRN. *H*^2^, broad sense heritability contributions of GRN (%). The green center line denotes the median value (50th percentile), and the green box contains the 25th to 75th percentiles of the dataset. The green whiskers mark the 5th and 95th percentiles. **i**, Radar plot showing the statistical significance of key cardiometabolic phenotype associations with GRN195 (top) and GRN122 (bottom). The significance of GRN–phenotype associations (−log_10_; *P* = 0–100) was calculated by aggregating GRN gene–level phenotype associations (Pearson correlation two-tailed *t*-test) corrected for the total number of STARNET GRNs (*n* = 135) and the number of genes in each GRN using the Benjamini–Hochberg procedure. Source data

Subclustering of scRNA seq data has in recent years efficiently been used to identify subclusters of cells in both healthy and diseased organs including the atherosclerotic arterial wall^4,9–13,17,23^. In disease states, however, only a fraction of genes in any given subcluster is responsible. In contrast, by identifying correct regulatory relationships among genes, GRNs provide mechanistic frameworks for how genes interact and function including pinpointing disease initiation and progression^24,25^. GRNs also provide key driver genes responsible for regulating their overall gene activities with a GRN as well as associations with underlying clinical phenotypes^26,27^. Using the STARNET study^5,18^, we recently provided a first framework of GRNs involved in promoting cardiometabolic disorders in relation to the progression of human atherosclerosis or coronary artery disease (CAD). To pinpoint putative atherosclerosis mechanisms and key disease driver genes as putative targets within the male and female carotid plaque subcellular clusters, we examined the enrichments of subcluster genes identified as being sex biased (Figs. 2–4) in 135 tissue-specific GRNs previously identified in STARNET^5^ (Figs. 5–7).

**Extended Data Fig. 5:**
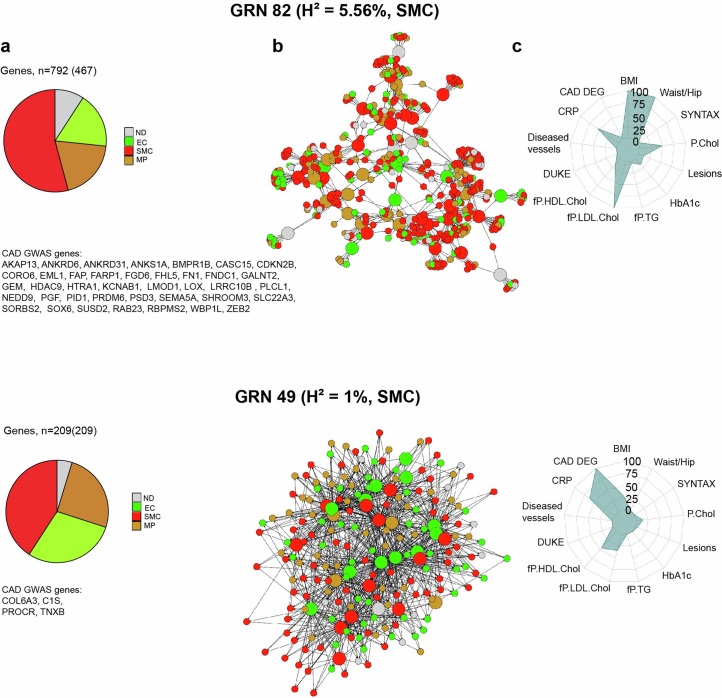
Enrichment of sex-diversified subcluster genes in human GRNs. 135 tissue-specific GRNs inferred from genotype and bulk RNAseq data of two arterial wall (atherosclerotic aortic root, and the non- or early atherosclerosis mammary artery and four metabolic (liver, skeletal muscle, subcutaneous fat and visceral abdominal fat) tissues obtained from 600 CAD patients of the STARNET study were inferred using block-wise WCGNA and the GENIE3 algorithms, as described^5^. The enrichments of sex-diversified subcluster genes in the SMC-specific GRN82 and GRN49 shown in Fig. 5a. **a**) Pie charts showing the relative cell type specificity of genes in GRN82 (top), GRN49 (bottom) according to the scRNA seq data (Methods). Below the pie charts, abbreviations of GRN82 (top) and GRN 49 (bottom) GWAS CAD candidate genes. **b**) GRN82 (top) and GRN 49 (bottom) color-coded according to the cell type specificity. Bigger size nodes are key driver genes. **c**) Radar plot showing statistical significance of key cardiometabolic phenotypes associations with GRN82 (top) and GRN 49 (bottom). The significance of GRN-phenotype associations ( − log10; P = 0–100) was calculated by aggregating GRN gene-level phenotype associations (Pearson correlation two tailed t-test) corrected for the total number of STARNET GRNs (n = 135) and the number of genes in each GRN using the Benjamini-Hochberg procedure. Source data

In SMCs, six arterial-wall GRNs were significantly enriched (−log_10_(false discovery rate (FDR) < 10%) in genes from sex-biased SMC subclusters (Fig. 5a and Extended Data Fig. 5a–c). Three GRNs, GRN82, GRN49 and GRN39, were enriched in genes from SMC subclusters representing both sexes (Fig. 5a). In contrast, the Synergy Between Percutaneous Coronary Intervention with Taxus and Cardiac Surgery (SYNTAX)-associated (Fig. 5b) and DUKE-associated (Fig. 5c) GRN177 was uniquely and strongly enriched in genes from the male-dominant SMC1 representing a contractile phenotype (Fig. 2). While this network was not enriched in genes associated with carotid symptoms in our study (Fig. 5d), it was significantly enriched in genes previously identified as being sex biased^28^ (Fig. 5e). The SMC-dominant (Fig. 5f,g) GRN177 contained 43 key drivers and 19 CAD genome wide association studies (GWAS) candidate genes (*ANKRD6*, *ARHGAP26*, *CNNM2*, *FNDC3B*, *GEM*, *HECA*, *MAP1B*, *MAP3K7CL*, *NBEAL1*, *NEDD9*, *NT5C2*, *PALLD*, *PDLIM5*, *PPP1R12B*, *PREX1*, *RAB23*, *RUNX2*, *SHROOM3*, and *SLC22A3*) and contributed to 2.16% of CAD *H*^2^ (Fig. 5g). Consistent with the phenotype of SMC1, GRN177 involved actin filament- and cytoskeletal-based biological processes (GO:0030029/36, Bonferroni-corrected *P* = 3.0705 × 10^−34^). To pinpoint male-specific modulators of GRN177 activity, we examined the expression pattern of top key drivers in male patients with carotid stenosis and during atherosclerosis progression in male mice with a human-like apoB100-containing lipoprotein profile (that is, the *Ldlr*^*−/*^^*−*^*Apob*^100/100^ mouse model^29^; Fig. 5h). In this fashion, *ILK* was identified as a potential target to modulate GRN177 activity specifically in males. Besides atherosclerosis severity scores (Fig. 5b,c), GRN177 was also enriched in genes associated with BMI and blood levels of LDL and HDL cholesterol (Fig. 5i).

### Integration of sex-biased MP clusters with human GRNs

Three arterial-wall GRNs, GRN33/GRN3 and GRN174, were strongly enriched in genes from the female-dominant lipid-laden inflammatory MP8 and the female-dominant immune-response-related MP3, respectively (Fig. 6a). In GRN3, present in the early-atherosclerotic mammary artery, 82 of its 115 genes overlapped with the 347 genes present in the late-atherosclerosis GRN33, suggesting that GRN3 represents an early version of GRN33. GRN33 was strongly enriched in genes associated with CAD severity (Fig. 6b,c) and, together with GRN174, significantly enriched in carotid symptom (Fig. 6d) and sex-biased (Fig. 6e) genes.

The lipid-laden, inflammatory TREM2^−^/TREM1^+^high role of MP8 (Fig. 3) was consistent with the overall function of GRN33 involving biological processes that change the state or activity of cells (for example, movement, secretion, enzyme production, gene expression and so on) in response to lipid or cytokine stimulus (GO:0033993/0034097, Bonferroni-corrected *P* = 4.8448 × 10^−41^). The MP-dominated GRN33 (Fig. 6f,g, upper panel) contained 33 key drivers, contributed to 1.14% of CAD *H*^2^ and harbored 10 CAD GWAS candidate genes (Fig. 6g; *CXCL8*, *LDLR*, *MAT2A*, *MCL1*, *OSM*, *PKD2L1*, *RASGEF1B*, *PKD2L1*, *SLC22A4* and *TRIB1*). To pinpoint key drivers that may be suitable female-specific modulators of GRN33, we examined its top key driver, *CEBPD*, which was shown to be upregulated in female versus male patients with carotid stenosis and to increase its expression levels during atherosclerosis progression in female *Ldlr*^−^^*/*−^*Apob*^100/100^ mice (Fig. 6h, upper panel). Consistent with its strong association with atherosclerosis severity (Fig. 6b,c), GRN33 was also highly enriched in genes differentially expressed in the arterial wall between CAD cases and controls and associated with BMI and blood levels of LDL and HDL cholesterol (Fig. 6i, upper panel).

GRN174 was predominately enriched in genes from the female-dominant MP3 (Fig. 6a), which played an immune-regulatory role (Fig. 3). Constituently, the entire GRN174 involved regulation of immune responses (GO:0050776, Bonferroni-corrected *P* = 1.2458 × 10^−140^). GRN174 had 27 key drivers, contributed 1.58% of CAD *H*^2^ and harbored 12 CAD candidate GWAS genes (Fig. 6g, lower panel; *ARHGAP15*, *CD300LF*, *CTSS*, *LINC00861*, *MAP3K1*, *NUP210*, *PLCB2*, *RUNX2*, *SCML4*, *SERPINA1*, *TBXAS1*, *ZNF831*). About 90% of the GRN174 genes represented MP gene activity (Fig. 6f,g, lower panels). During atherosclerosis progression in female *Ldlr*^−/−^*Apob*^100/100^ mice, the expression levels of the top key driver, *LYZ*, increased and *LYZ* was upregulated in female versus male carotid plaques (Fig. 6h, lower panels) suggesting that it may be a suitable target to modulate GRN174 activity specifically in females. GRN174 was also strongly enriched in genes associated with blood levels of LDL cholesterol and C-reactive protein (CRP) (Fig. 6i, lower panel).

### Integration of sex-biased EC clusters with human GRNs

Three arterial-wall GRNs (GRN113, GRN122 and GRN195) were strongly enriched in genes from two sex-biased carotid plaque EC subclusters both identified in the vasa vasorum: GRN113/GRN195, in genes from the male EC1 subcluster (Fig. 7a) related to angiogenesis and T cell-mediated cytotoxicity (Fig. 4e), and GRN122 in genes from the female EC2 cluster (Fig. 7a) related to endoMT and endICLT (Fig. 4f). GRN195 and GRN122 were strongly enriched in genes associated with SYNTAX (Fig. 7b) and Duke (Fig. 7c) scores, respectively, and in carotid stenosis symptom (Fig. 7d) and sex-biased (Fig. 7e) associated genes.

Like GRN3, which represented an early atherosclerosis version of GRN33 in MPs (Fig. 6), GRN113 represented an early version of GRN195 in ECs manifested by 73 of its 141 genes being also present in the late-stage atherosclerosis GRN195. With 198 genes and 20 key driver genes, GRN195 contributed 0.6% of CAD *H*^2^ and contained 7 CAD GWAS candidate genes: *FLT1*, *NOS3*, *PECAM1*, *PLCG2*, *PLEKHG1*, *TEK* and *THSD7A* (Fig. 7g, upper panel). Like EC1, GRN195 was involved in vasculature development or angiogenesis (GO:0001944, Bonferroni-corrected *P* = 2.1167 × 10^−30^). Approximately 90% of GRN195 genes represented EC gene activity (Fig. 7f,g, upper panels). According to the expression pattern during atherosclerosis progression in male *Ldlr*^−*/*−^*Apob*^100/100^ mice and the levels in male carotid plaques, the top key driver, *PLVAP*, constitutes a plausible target to modify the activity of GRN195 in males (Fig. 7h, upper panels). In addition to the SYNTAX score, GRN195 was also highly enriched in genes differentially expressed in the arterial wall between CAD cases and controls (Fig. 7i, upper panel).

GRN122 contributed to a remarkable 4.10% of CAD *H*^2^ and contained 16 CAD GWAS candidate genes: *ARHGAP15*, *C2*, *CFAP161*, *CTSS*, *EDEM2*, *MITF*, *NPC1*, *PID1*, *PLCB2*, *SCARB1*, *SERPINA1*, *TARID*, *TBXAS1*, *VAMP8*, *ZC3H12D* and *ZNF831* (Fig. 7f,g, lower panels). According to the expression pattern during atherosclerosis progression in female *Ldlr*^*−/−*^*Apob*^100/100^ mice and the levels in female carotid plaques, the top key driver, *CTSB*, constitutes a plausible target to modify the activity of GRN122 in females (Fig. 7h, lower panels). In addition to the Duke score (Fig. 7c), GRN122 was strongly enriched in genes associated with BMI and blood levels of total and LDL cholesterol (Fig. 7i, lower panel). According to our annotation using the carotid scRNA-seq data, the 766 genes of GRN122, whereof 30 key driver genes were ∼90% of MP origin (Fig. 7f,g, lower panels), involved regulation of immune responses (GO:0050776, Bonferroni-corrected *P* = 2.1156 × 10^−174^). As such, the strong enrichment of this network with EC2 genes may appear unexpected. However, this may at least in part be explained by the fact that a majority of the predominantly female EC2 cells undergo either endoMT or endICLT. Notably, GRN122 was also enriched in genes from several immune-regulatory MP subclusters (Fig. 6a).

### Reproducibility and clinical validation of the sex-diverse GRNs

**Extended Data Fig. 6:**
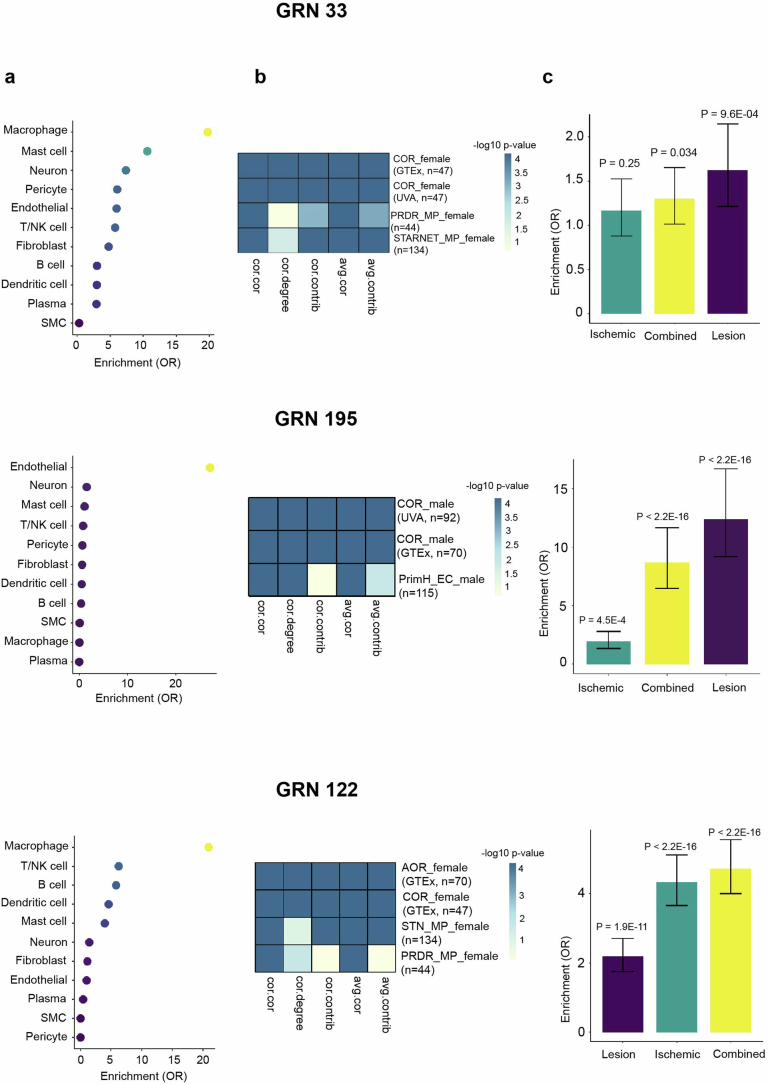
Independent evaluation of GRN33, GRN195 and GRN122 cell-specificity, reproducibility and roles in atherosclerotic cardiovascular disease. **a**) GRN33 (top), GRN195 (middle) and GRN122 (bottom) cell-type specificity according to the enrichment of cell-type determined genes in scATAC-seq data obtained from 41 human coronary artery tissue samples (Methods). Dot plots show odds ratio of enrichment of GRN33, GRN195 and GRN122 module genes for marker genes determined from integrated snATAC-seq and scRNA-seq profiles obtained from atherosclerotic coronary artery tissues^30^ (Methods). **b**) GRN33 (top), GRN195 (middle) and GRN122 (bottom) reproducibility according to NetRep^31^. The original GRN33 connectivity in bulk RNAseq data from the atherosclerotic aortic wall in STARNET was compared with that of two bulk coronary (UVA and GTEx), primary blood MP cell (STARNET)^5^, and one additional primary blood MP (NGS-PREDICT, Ma et al., unpublished) RNAseq datasets filtered for female donors. The original GRN195 connectivity in bulk RNAseq data from the atherosclerotic aortic wall in STARNET was compared with that of two coronary (UVA and GTEx) and one human aortic endothelial cell (HAEC) RNAseq datasets, the latter filtered for male donors^57^. The original GRN122 connectivity in bulk RNAseq data from the atherosclerotic aortic wall in STARNET was compared with that of bulk coronary and aorta (GTEx), primary blood MP cell (STARNET)^5^, and one additional primary blood MP (NGS-PREDICT, Ma et al., unpublished). RNAseq datasets filtered for female donors. ‘cor.cor´ measures the concordance of correlation structures; ‘cor.degree´ measures the concordance of the weighted degrees; ‘cor.contrib´ measures the concordance of node contributions; ‘avg.cor’ measures the average magnitude of correlation coefficients and ‘avg.contrib´ measures the average magnitude of node contributions. The bar shows log^−10^ *P*-values. P-values were adjusted for multiple testing using the Benjamini & Hochberg method. **c**) Barplots showing the odds ratio (OR) for GRN33 (top), GRN195 (middle) and GRN122 (bottom) enrichments in coronary artery differential expressed genes comparing (1) lesions (n = 28) versus non-lesions (n = 26), (2) ischemic (n = 38) versus non-ischemic (n = 23) heart explants, and (3) combined lesion and ischemic (Methods). The Fisher test was performed on overlapping gene sets, the p-values shown represent the Fischer test (two-tailed) p-values and error bars represent 95% confidence intervals. Data represent Mean ± SEM. Source data

Among the GRNs enriched in sex-biased carotid plaque subcellular clusters, the female MP GRN33 and the male EC GRN195 stood out when they first appeared in early atherosclerosis forms in the mammary artery, as GRN3 and GRN113, respectively. In addition, the female MP GRN122 was involved in the regulation of endoMT and endICLT–EC transitions believed to be critical for plaque erosion in females^28^. We therefore sought to examine the cellular origin, reproducibility and clinical implications of these GRNs using six independent coronary artery single-nucleus assay for transposase-accessible chromatin using sequencing (snATAC-seq), bulk RNA-seq datasets, and EC- and MP-specific RNA-seq datasets. The cellular origins of all these GRNs were robustly confirmed in coronary lesion snATAC-seq and scRNA-seq data^30^ (Extended Data Fig. 6a). To examine the reproducibility of GRN33, GRN195 and GRN122, we applied the NetRep application^31^ (Extended Data Fig. 6b). The female-dominant GRN33 and GRN122 were robustly replicated in the two coronary artery bulk datasets filtered for females and generally in two human primary MP-specific RNA-seq datasets obtained from females in the Next Generation Sequencing to Predict Risk Events of Disease in the Coronary Tree (NGS-PREDICT) and STARNET studies. Similarly, the male-dominant EC GRN195 was robustly replicated in the two coronary bulk datasets filtered for males as well as in primary human arterial ECs obtained from male donors (Extended Data Fig. 6b, middle panel). In addition, all three GRNs were enriched in differentially expressed genes (DEGs) identified in (1) human coronary arteries between lesion and non-lesion samples, (2) ischemic versus nonischemic patients with CAD and (3) the combination of both (Extended Data Fig. 6c). In summary, these independent evaluations confirm the reproducibility of GRN33, GRN122 and GRN195 and underscore their sex biases and roles across different forms of atherosclerotic cardiovascular disease.

### Validation of GRN195 in human aortic ECs

**Fig. 8:**
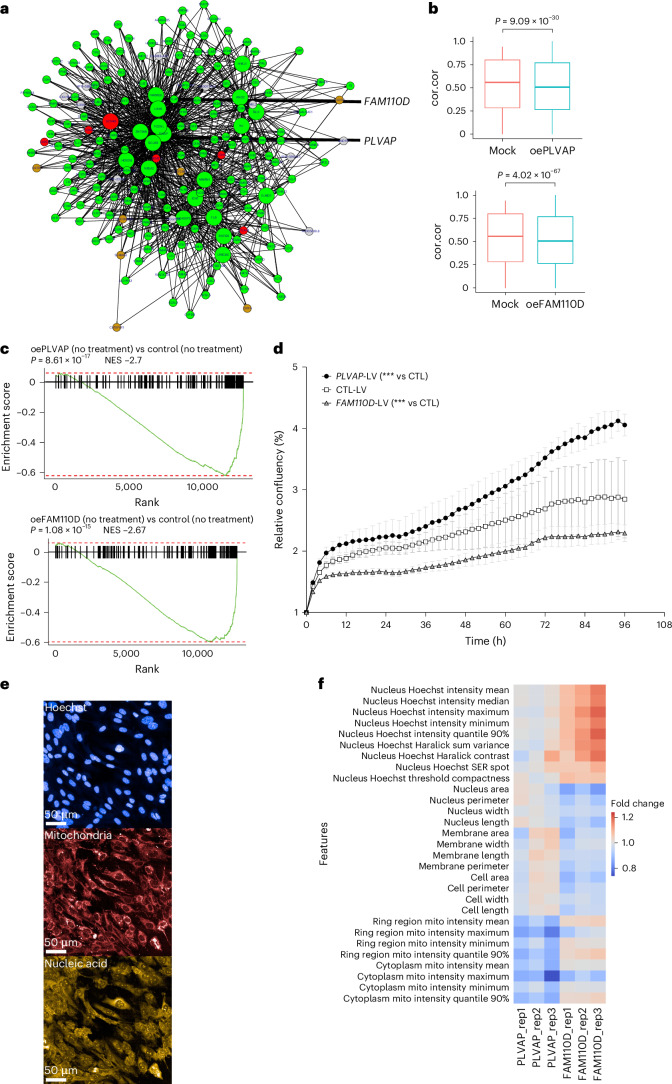
Experimental validation of GRN195 by overexpressing its top key drivers *PLVAP* and *FAM110D* in HAECs. The top key drivers of GRN195, *PLVAP* and *FAM110D*, were overexpressed in HAECs followed by RNA-seq. **a**, *PLVAP* and *FAM110D* are highlighted in the color-coded GRN195 network according to the cell type specificity. **b**, Average pair-wise correlation of GRN195 coding genes across different conditions, comparing Mock, oePLVAP and oeFAM110D groups with four replicates each. Mock, cells with lenti virus without construct, i.e., acts as control; oePLVAP, primary cells transduced with lentiviral vectors to overexpress *PLVAP* gene; oeFAM110D, primary cells transduced with lentiviral vectors to over express *FAM110D* gene; cor.cor, concordance of correlation structure. In the box plot, the center line denotes the median value (50th percentile), and the box contains the 25th to 75th percentiles of the dataset. The statistical test was conducted using *t*-test (two-sided). No adjustment was made and there was no multiple comparison. **c**, GSEA of RNA-seq data of HAECs overexpressing *PLVAP* and *FAM110D*. The NES indicate the direction and strength of gene set enrichment. The statistical test used was GSEA, which uses a one-sided test to assess whether a predefined gene set is significantly enriched at either the top or bottom of the ranked gene list. *P* values were calculated using permutation testing, and multiple comparisons were adjusted using the FDR correction. **d**, Relative proliferation in the *PLVAP* group compared with the control (CTL) group shows a significant increase, while the *FAM110D* group shows a significant decrease relative to CTL. Statistical analysis was conducted using repeated-measures ANOVA (two sided) Dunnett’s multiple comparison test, *P* < 0.0001. Data represent the mean ± s.d. ***, *P* < 0.0001. **e**, Representative images of cell painting showing nuclear (blue), mitochondria (red) and nucleic acid (yellow) stains at day 6 in LV-overexpressing HAEC cells. Scale bar = 50 µm. A total of 4 wells per group including 10 regions per well were imaged (*n* = 40 images per group) with two biological replicates. **f**, Heatmap illustrating the effect of LV-*FAM110* and LV-*PLVAP* overexpression on cell phenotypic features in HAECs at day 6 detected by cell painting. A total of 4 wells per group including 10 regions per well were imaged (*n* = 40 images per group) with Opera Phenix Plus High Content Screening System, and the cell morphological signature was analyzed by Harmony analysis software. Two independent experiments were performed. Heatmap values show the fold changes over the LV-EGFP control vector. mito, mitochondria. Source data

**Extended Data Fig. 7:**
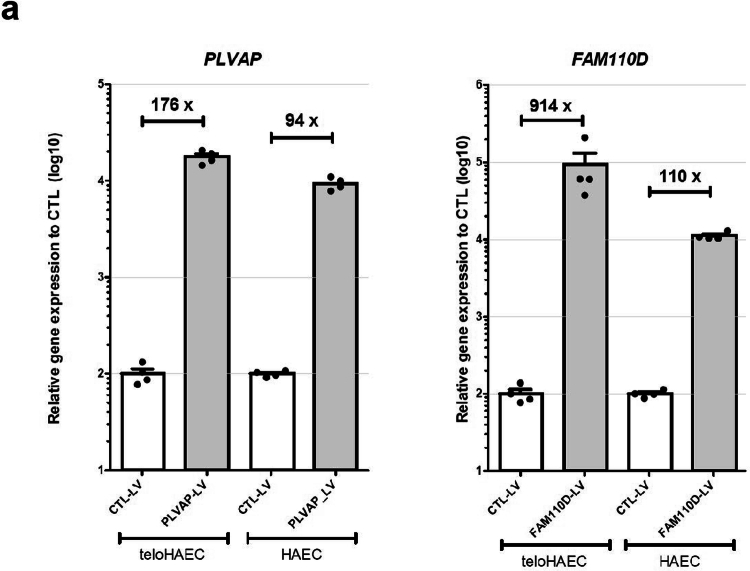
Relative gene expression of PLVAP and FAM110D in human aortic ECs. **a**) Relative gene expression of PLVAP and FAM110D in Telo-HAECs and HAECs compared to control lentivirus transduced cells. The qPCR was performed in four replicates. Data represent Mean ± SEM. Source data

To experimentally validate GRN195, we targeted its top drivers, *PLVAP* and *FAM110D*^5^, in HAECs (Fig. 8a). Given the low basal expression levels of *PLVAP* and *FAM110D*, we opted to overexpress these genes, followed by RNA-seq (Extended Data Fig. 7a) and cell painting analyses. *PLVAP* and *FAM110D* overexpression both significantly reduced the overall connectivity of GRN195 (Fig. 8b, top and bottom panels). Consistently, gene set enrichment analysis indicated a negative normalized enrichment score (NES) (Fig. 8c, top and bottom panels).

**Extended Data Fig. 8:**
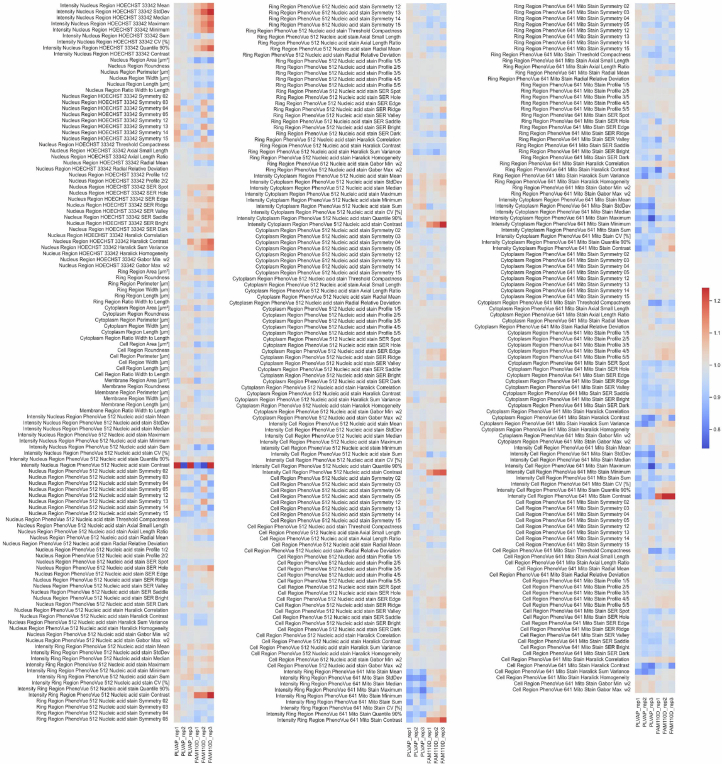
Cell painting to identify phenotypic features associated with the FAM110D and PLVAP overexpression. Over 300 basic morphology, STAR morphology, SER texture and Haralick features were measured from fluorescent stained samples of LV-FAM110D and LV-PLVAP overexpressing HAEC cells. Source data

Cell painting assays were used to study the phenotypic features associated with the overexpression of *FAM110D* and *PLVAP* (Fig. 8e). Over 300 basic morphology, symmetry, threshold compactness, axial or radial (STAR) morphology, spots, edges and ridges (SER) texture and Haralick features were assessed in fluorescent stained HAECs (Extended Data Fig. 8a). Compared with controls, cells overexpressing *FAM110D* showed increased nuclear intensity features and reduced nuclear areas, suggesting enhanced chromatin condensation consistent with reduced GRN195 activity (Fig. 8e). In addition, cell and membrane area reductions were observed, consistent with RNA-seq data showing downregulation of cell adhesion genes in both *FAM110D*-overexpressing HAECs and telomerase-immortalized human aortic endothelial cells (Telo-HAECs) (Supplementary Tables 56, 57 and 60). Genes involved in ribonucleoprotein complex biogenesis and cell cycle progression were upregulated. Functional assays confirmed a significant decrease in EC proliferation in *FAM110D*-overexpressing cells compared with controls (Fig. 8d).

*PLVAP* overexpression resulted in decreased mitochondrial staining intensity, as detected by cell painting (Fig. 8e,f). This corresponded with RNA-seq data showing a trend toward downregulation of oxidative stress response genes (Supplementary Tables 58, 59 and 61). In addition, cell-cycle-related genes were upregulated, and EC proliferation was increased in *PLVAP*-overexpressing cells (Fig. 8d). These data indicate that *PLVAP* overexpression is associated with increased EC proliferation.

Collectively, overexpression of *FAM110D* and *PLVAP* in HAECs specifically impacts GRN195 gene activity along with several atherosclerosis-relevant EC phenotypes including chromatin condensation, adhesion, mitochondrial activity and proliferation.

## Discussion

Through deep single-cell sequencing of 7,690 cells from human carotid plaques, we show that sex differences are nearly absent in major cell types, but are commonly observed in subcellular clusters of these cells. In fact, 6 out of 9 SMC, 6 out of 8 MP and 3 out of 5 EC subcellular clusters were significantly sex biased. By integration with 135 arterial-wall- and metabolic-tissue-specific GRNs^5^, we could further pinpoint arterial-wall pathological mechanisms, clinical contexts including CAD heritability contributions and key disease driver genes for several of the sex-biased subcellular clusters: in the advanced atherosclerotic arterial wall, the male- and SMC-dominant GRN177 linked to CAD severity involving regulation of contractile SMC functions and the male vasa vasorum EC-specific GRN195 involving angiogenesis and T cell-mediated cytotoxicity were identified. In females, the early atherosclerotic arterial-wall MP-dominant GRN174 involving regulation of immune responses was identified together with the advanced atherosclerosis arterial-wall MP-dominant GRN33 involving lipid-laden TREM2^−^/TREM1^+^-high and inflammatory macrophages and the EC and MP GRN122 involving endoMT. Of these GRNs, the female GRN33 and male GRN195 with early atherosclerosis counterparts (that is, GRN3 and GRN113, respectively) as well as the female GRN122 were successfully replicated and clinically validated in six independent human-cell-specific and bulk arterial-wall RNA-seq datasets. In addition, the presence and function of the male GRN195 was explicitly verified in cultured HAECs by overexpressing its top key drivers, *PLVAP* and *FAM110D*.

The gene contents of the carotid plaque subcellular clusters identified in our study did not only capture but, due to deeper sequencing provided by Smart-Seq2 (>7,000 genes per cell), also substantially extended our current understanding of the cellular transformation taking place in human atherosclerosis^4,8–14,17,23,29,32–34^. In SMCs, the SMC1–SMC9 trajectory captured the overall transformation of contractile SMCs into endothelial-like cells during later stages of atherosclerosis^9,10^. More specifically, besides the native athero-protective contractile^12^ (SMC1 and SMC2) and osteogenic-like (SMC8) SMCs, pro-atherogenic MP-like^11^ (SMC7), endothelial-like (SMC9) and fibrochondrocyte-like (SMC3–SMC5) forms of SMCs were identified. Of these, SMC1, SMC4, SMC5 and SMC9 were significantly male biased whereas SMC3 and SMC8 were significantly female biased (Fig. 2). For the MP subclusters, resident-like (MP1), dendritic-like (MP2 and MP3), SMC-like (MP4), lipid-poor pro-inflammatory^16^ (MP7 and MP8) and lipid-laden TREM2-expressing^17^ (MP5 and MP6) MPs were identified. Of these, MP1 and MP7 were significantly male biased whereas MP2, MP3, MP5 and MP8 were significantly female biased (Fig. 3). ECs found in our study originated from two vascular beds: the vasa vasorum within the carotid plaques and those facing the carotid artery lumen. The vasa vasorum EC subclusters were involved in angiogenesis and T cell-mediated cytotoxicity (EC1, male dominant)^34^, endMT (EC2, female dominant)^35^ and endICLT (EC3)^14^. The carotid-lumen-related ECs were either progenitor cells (EC4, female dominant)^22^ or mature ECs mainly involved in extracellular matrix organization (EC5)^22^. Overall, these sex-biased subcellular clusters are consistent with reported differences in carotid stenosis etiology and clinical manifestation between males and females.

Although clustering of scRNA-seq data has proven critically important to delineate subtypes of vascular cells promoting atherosclerosis, scRNA-seq data have yet to prove useful for the inference of biological co-expression networks. Critical components of ‘omics datasets to allow co-expression network inference are (1) gene (RNA) expression data reflecting the full spectra of variation associated with pathophysiology including lowly-expressed genes and (2) gene expression data coupled with genetic diversity representing at least hundreds of individuals. Currently, scRNA-seq datasets (including the current) do not fulfill these criteria. Moreover, given that the primary goal of scRNA-seq analysis is to define disease-relevant subtypes of cells, subsequent network inference within these subtypes is simply untenable as the subtype data lack sufficient depth. Moreover, although at least one co-expression network method based on single-cell data has been proposed^36^, the lack of genetic diversity still prevents the inference of directional co-expression networks (that is, GRNs), which in turn prevents the identification of key disease drivers, heritability contributions and causal inference of networks with clinical phenotypes. On the other hand, the human cardiometabolic-focused, multi-omics STARNET datasets, obtained from hundreds of living patients with and without obstructive coronary atherosclerosis, meet all the above criteria. Hence, by integrating STARNET GRNs, we could provide key drivers, clinical contexts, heritability contributions and pathophysiological mechanisms to the sex-biased subcellular clusters identified in human carotid plaques. Using this approach, we successfully identified several largely cell-type-specific GRNs (Figs. 5–7) representing sex-diverse mechanisms of atherosclerotic cardiovascular disease important for the development of carotid stenosis.

The late-atherosclerotic arterial-wall GRN33 and its predecessor in the early-atherosclerotic mammary artery, GRN3, were exclusively enriched in genes from the female-dominant lipid-associated and inflammatory-like MP8 subcluster (Fig. 3d). The genes of GRN33 and its top biological processes, response to lipid (GO:0033993) and response to cytokines (GO:0034097), align well with the role of the MP8 subcluster genes and what is known about TREM1^+^/TREM2^−^ residing deeper in the lipid core as an important component of the intraplaque atherogenic inflammatory response enriched in patients who recently experienced cerebrovascular events. In these MPs, PLIN2^hi^/TREM1^hi^ represent a terminal state showing signatures of lipid handling, inflammation and apoptosis^37^. Besides TREM1, inflammatory genes such as *TNF* and *IL1B* as well as chemokines such as *CCL20*, *CXCL1*, *CXCL2*, *CXCL3* and *CXCL8* are activated in these MPs. The TREM2^hi^ macrophage may transition toward an inflammatory PLIN2^hi^/TREM1^hi^ lipid handling, inflammation and apoptosis phenotype by VCAN–TLR2 ligand receptor pairing^13^. We show that GRN33 and its top key driver, *CEBPD*, known for its importance in regulating genes involved in immune and inflammatory responses as well as activation and/or differentiation of macrophages, play a critical role in regulating TREM2^−^/TREM1^+^high macrophages specifically in females. This notion is further underscored by the robust evaluation of GRN33 in six independent arterial-wall and cell-specific female datasets including its macrophage origin and role in coronary ischemia and lesion formation.

Under the influence of certain stimuli such as transforming growth factor beta (TGFb), some ECs undergo endoMT—a process in which the adhesion between ECs is weakened and is replaced by increased migratory capabilities resulting in ECs resembling mesenchymal cells. If the stimulus is lost, the endoMT process may reverse. Depending on the degree of EC–EC adhesion loss, endoMT causes ECs to migrate either in a chain of cells (low loss) or individually (high loss). The latter has been shown to cause carotid plaque erosion^38^, in which the gap between the migrating ECs exposes the collagen surface of the plaque directly to the bloodstream leading to the formation of a thrombus. Plaque erosion leading to thrombosis has been reported as a complication of carotid stenosis^35^ partially in females. Importantly, endoMT, such as SMC phenotypic switching, is not a one-way transition but rather based on stimulus and causes ECs to transition toward either a more athero-protective phenotype (that is, an ACTA2^+^ phenotype) or a atherosclerosis-prone migratory phenotype (that is, an FN1^+^ phenotype)^39^. The athero-protective role of ACTA2^+^ ECs in the atherosclerotic lesion is believed to involve compensating the loss of SMCs maintaining the fibrous cap preventing plaque rupture^40^. In this way, endoMT is a critical link between inflammation, impaired hemodynamics and tissue remodeling involved in the plaque formation^35^. In our study, we found that 84% of the cells in the vasa vasorum subcluster EC2, representing the ACTA2^+^ endoMT phenotype along with antigen-presenting genes, and 94% of the cells in the carotid lumen subcluster EC4, representing the FN1^+^ endoMT phenotype, were isolated from females. In addition, not only EC2 but also EC4 genes were enriched in GRN122 (Fig. 7a), a network containing many antigen-presenting genes including the top key drivers *HLA-DPA1*, *HLA-DQA1*, *HLA-DOA* and *HLA-B* implying that this network plays a critical role in regulating endoMT and inflammation. This notion is further underscored by GRN122 top biological processes, regulation of immune responses (GO:0050776) and cell activation (GO:0001775).

Of the vasa vasorum ECs forming EC1, 85% were isolated from male carotid plaques (Fig. 4d). GRN195 and its early predecessor, GRN113, were exclusively and strongly enriched in genes from EC1 (*n* = 170 of the 198 genes in GRN195; Fig. 7a). Of the 20 top key driver genes of GRN195 (Fig. 7g), 10 were related to angiogenesis, such as *BCL6B* (vascular homeostasis), *HYAL1* (angiogenesis), *PODXL* (endothelial-to-mesenchymal transition) and *PLVAP* (endothelial transcytosis). This is noteworthy because, in response to hypoxia, atherosclerotic plaque angiogenesis typically originates in the vasa vasorum^41^ resulting in immature neo-vessels that are prone to rupture^42,43^. Together with large lipid cores, a typical male carotid stenosis feature, immature neointimal vasa vasorum has been shown to be more prone to cause intraplaque hemorrhages enlarging the necrotic core destabilizing the plaque^44^. For these reasons, we decided to further evaluate male-dominant GRN195: first, in six independent male arterial-wall and cell-specific datasets in which GRN195 was readily reproduced including its EC origin and importance for both coronary lesion formation and ischemic events (Extended Data Fig. 6, middle panel); second, in cultured HAECs by overexpressing GRN195 top key drivers, *PLVAP* and *FAM110D*, we show that GRN195 indeed represents a coherent function in ECs involving angiogenesis. Thus, GRN195 with its top key drivers *PLVAP* and *FAM110D* may be suitable targets for regulating neovascularization of the vasa vasorum particularly in males to prevent carotid plaques from destabilizing and ensuing clinical symptoms.

Our study has limitations. Our deep sequence carotid stenosis data, obtained from thousands of cells, was generated from the relatively small number of 15 study participants, 7 female and 8 male patients. Noteworthily, however, our sample size is in the higher bracket of participant sizes compared with those in published scRNA-seq studies in humans thus far^4,10,13,14,17^. Moreover, although we sought to match the 7 female and 8 male patients (Supplementary Tables 1 and 2), the female group was, on average, 4–6 years older, probably reflecting the fact that women before menopause to some degree are protected from atherosclerosis, thus developing clinical manifestations later than men. In addition, FACS sorting, as an integral part of the Smart-Seq2 protocol, probably underestimates rare cell types not recognized by the selected antibodies, thus failing to identify plausible sex diversities in rarer cell types such as T cells and pericytes. The GRNs in our study were originally identified in patients with CAD, not those with carotid stenosis. Although the atherosclerotic processes leading to clinical events such as stroke are mostly common between CAD and carotid stenosis, the sex-diverse subcellular clusters we identified in the carotid plaques may to some degree have other clinical and pathophysiological implications not captured by GRNs originally identified in the STARNET study^5^.

By generating a deep scRNA-seq dataset of human carotid plaques, our study shows that cellular subtypes of carotid plaques representing key atherosclerosis processes are highly sex biased. Moreover, by integration with a human framework of GRNs, we identified several arterial-wall, largely cell-specific GRNs that provide clinical contexts and pathophysiological mechanisms to these sex-biased atherosclerotic cell subtypes including GRN122, GRN33 and GRN195 that were independently verified. Overall, our findings call for further sex-stratified studies of carotid stenosis with the purpose of better tailoring diagnostics and therapies for male and female patients with life-threatening symptoms from atherosclerotic cardiovascular disease.

## Methods

### Patients with carotid stenosis and isolation of single cells

Informed consent was obtained from 15 carefully characterized patients (8 males and 7 females) eligible for carotid endarterectomy because of significant stenosis (>70%) in the proximal part of their internal carotid arteries, and these patients were included in the study (Institutional Review Board number 289/T-12; Research Ethics Committee of the University of Tartu) (Supplementary Table 1). No compensation was provided to the participants of the study. Patients were characterized as asymptomatic (*n* = 7, no clinical signs of brain ischemia) or symptomatic (*n* = 8, clinical signs of brain ischemia such as minor stroke, transitory ischemic attacks, retinal infarction or amaurosis fugax (brief visual disturbance). None of the female patients in our study reported using (or have been using) hormone therapy. Once collected, the entire carotid plaques were immediately processed in Hanks’ Balanced Salt Solution (HBSS) solution with 10 mg ml^−1^ of Collagenase type I and II and Elastase (Sigma) for 1 h at room temperature followed by smooth mechanical disruption to obtain a single-cell suspension. After centrifugation at 300 × *g* at 20 °C for 5 min, the supernatants were removed and cell pellets resuspended in PBS buffer supplemented with 0.5% bovine serum albumin, 2 mM EDTA and 25 mM HEPES. To enrich for SMCs, ECs and immune cells, the human cell suspensions were labeled with anti-PDGFRβ, anti-CD31/CD144 and anti-CD45 (R&D Systems), respectively.

### The *Ldlr*^−/−^*Apob*^**100/100**^ mouse model

Unlike the unphysiological lipid levels in plasma of single-gene targeted atherosclerosis mouse models (for example, the apoE and Ldlr knockout, and transgenic PCSK9 models), the genetic modifications of the *Ldlr*^−/−^*Apob*^100/100^ mice^45^ produce a human-like plasma lipoprotein profile with most of its plasma cholesterol in apoB100-containing LDL particles at human-like plasma cholesterol concentrations (that is, 200–250 mg dl^−1^). Consequently, the *Ldlr*^−/−^*Apob*^100/100^ mouse model develops advanced atherosclerotic lesions on a chow diet. In this model, we previously quantified atherosclerotic lesions en face in pinned-out aortas at the ages of 20, 30, 40, 50 and 60 weeks^45^. In our study, we used 46 independent *Ldlr*^−/−^*Apob*^100/100^ mice bred on 100% C57Bl6/J genetic background divided into 4 baseline (non-atherosclerotic, 20 weeks of age or less), 9 early-atherosclerotic (30–40 weeks of age) and 10 advanced-atherosclerotic (>50 weeks of age) pairs of aortic arches isolated from 2 independent mice. The numbers of mice used at baseline were as follows: 8 mice (2 female, 6 male); early stage: 18 mice (6 female, 12 male) and advanced stage: 20 mice (10 female, 10 male).

### Single-cell isolation from the aortic arch of *Ldlr*^−/−^*Apob*^**100/100**^ mice

All mouse experiments were carried out in accordance with Swedish and Finnish legislation and local guidelines and regulations for animal welfare and were approved by the Animal Research Ethics committee in Linköping, Sweden (dnr729) and the National Experimental Animal Board of Finland. No more than 6 mice were housed in standard, single ventilated cages with 12-h light–dark cycles, given ad libitum access to water and fed a chow diet. *Ldlr*^−/−^*Apob*^100/100^ mice^45^ bred onto a C57Bl6/J background were euthanized by cervical dislocation and perfused with PBS. The aortic arch was dissected from the aortic root to the third rib, cleaned from the adventitia and immediately placed into ambient PBS solution (Dulbecco’s Phosphate-Buffered Saline (DPBS), Thermo Fisher Scientific). To generate sufficient cells, two aortic arches were submitted to the first incubation in HBSS from Thermo Fisher Scientific with 2% BSA (Sigma), supplemented with 10 mg ml^−1^ of Collagenase type I, II and IV and 2 mg ml^−1^ of Elastase (all from Sigma) at 37 °C with horizontal shaking at 500–800 rpm for 10 min to separate ECs. For further enzymatic dissociation, the remaining aortic arch tissues were cut into smaller pieces and incubated in HBSS solution with 10 mg ml^−1^ of Collagenase type I and II and Elastase (Sigma) in three cycles followed by pipetting in 20-min intervals. The cell suspensions were then sequentially filtered through 70-μm and 40-μm cell strainers. To maximize cell retention, the 70-μm cell strainer was additionally washed with 5 ml of DMEM (Thermo Fisher Scientific). After centrifugation at 300 × *g* at 20 °C for 5 min and removal of the supernatant, cell pellets were resuspended in FACS buffer (PBS supplemented with 0.5% BSA, 2 mM EDTA, 25 mM HEPES). To allow FACS into three main cell populations, antibodies conjugated with fluorophores for ECs (anti-CD31-PE; Miltenyi Biotech, catalog number 130-102-608, 1/200), immune cells (anti-CD45-APC-CY7; BioLegend, catalog number 103116, 1/200) and SMCs (anti-CD140b-APC; R&D Systems, catalog number 12-1402-81, 1/200) were added to the suspensions together with Calcein Green AM (Thermo Fisher Scientific) to select viable cells. The labeled suspensions were further diluted with FACS buffer and placed on ice.

### FACS

To ensure enrichment and capture of viable and key atherosclerosis cells, the Calcein Green and antibody-stained cell suspensions were first processed using a FACS Melody cell sorter equipped with a 100-μm nozzle and then deposited into 384-well plates containing 2.3 μl lysis buffer (0.2% Triton X-100, 2 U ml^−1^ RNase inhibitor, 2 mM dNTPs, 1 μM Smart-dT30VN, ERCC 1:4 × 104 dilution). To maximize yield, we first used a generous gate for forward scatter area/side scatter area (FCS-A/SSC-A, linear scale) excluding events with low values containing cell debris and red blood cells. Next, doublet discrimination was implemented using FCS-A/FSC-height and SSC-A/SSC-height including a generous threshold for the distance of events from the diagonal line to prevent possible bias toward round-shaped cells. Last, cells passing the first two criteria were selected for Calcein Green AM (Thermo Fisher Scientific) and then sorted for the cell-specific antibody (Miltenyi Biotech, that is, for SMCs: anti-CD140b-APC, REAfinity (catalog number 130-121-128, 1/200); for ECs: anti-CD31-PE (catalog number 130-119-142, 1/200) and anti-CD144-APC (VE-Cadherin), REAfinity (catalog number 130-126-010, 1/100); and for immune cells: anti-CD45-PerCP (catalog number 130-113-682, 1/200) into individual wells of 384-well plates. To ensure correct gating, samples stained with single or no antibodies were used as negative controls. During sorting, plates were always kept at 4 °C and thereafter immediately stored at −80 °C (Supplementary Fig. 1).

### Smart-Seq2 single-cell library preparation and sequencing

Single-cell cDNA libraries were prepared using the Smart-Seq2 protocol, as described^19^. In brief, single-cell mRNA was transcribed into cDNA using oligo(dT) primers and SuperScript II reverse transcriptase (Thermo Fisher Scientific) and amplified by PCR for 24–25 cycles. Next, cDNA samples with sufficient quality according to DNA High Sensitivity chip analysis in a 2100 Bioanalyzer (Agilent Biotechnologies) were fragmented, tagged (tagmented) and indexed using Tn5 transposase and the Illumina Nextera XT index kits (Set A–D). Indexed cDNA libraries were pooled and sequenced using a HiSeq3000 (Illumina) with a dual-indexing and single-end, 50-base-pair (bp) read protocol.

### scRNA-seq data preprocessing

Raw sequences were demultiplexed and converted into FASTQ files using the Illumina bcl2fastq pipeline with default settings. The reference mouse and human genomes, GRCm38 (mm10) and GRChg38, applying the TopHat2 with Bowtie2 option were used for mapping, as described^46,47^. Alignments in the resulting BAM files were used for sorting. The Samtools software was used to remove duplicate reads and Feature Counts from the Subread package^48^ to calculate raw read counts. The 92 ERCC RNA included in the lysis buffer was used to ensure accurate mapping and as technical controls for raw read counts. ENSEMBLE annotation was performed using the org.mM.eg.db and org.Hs.eg.db packages (version 3.14.0) provided in the R software.

### scRNA-seq clustering

Combined cell raw read counts in gene expression matrixes were analyzed using the Seurat pipeline^20^. Libraries with abnormally high (≥750,000 suggesting possible cell doublets) or low (≤50,000) read counts or high relative content of mitochondrial or ERCC genes (≥10%) were removed. Genes expressed in <3 cells, or with a cumulative count <300 reads, were considered absent. The 2,000 most variable genes were used to calculate principal components of variation. Dimension reduction plots were visualized using the uniform manifold approximation and projection (UMAP) function^49^. The graph-based clustering approach with the *K*-nearest-neighbor graph from the Seurat pipeline was used for clustering. Gene ontology enrichment was performed using the GENEONTOLOGY platform (https://geneontology.org/). GRNs were reproduced from the STARNET database (http://starnet.mssm.edu/)^5^ using the network package in the R software (https://CRAN.R-project.org/package=network). Statistical significance was assessed using the Wilcoxon rank sum test. Within each cell type (SMCs, MPs and ECs), subcellular cluster genes were defined as those that were differentially expressed in a particular subcluster in relation to all other subclusters (that is, background) of a given cell type. The male-to-female proportion of cells within major cell types was statistically analyzed using the chi-square (*χ*^2^) test that measures how a model compares with actual observed data. It compares the size of any discrepancies between the expected results and the actual results, given the size of the sample and the number of variables in the relationship. To calculate an expected count for a cell, compute the product of the ‘row total’ and the ‘column total’ for the row and column containing that cell, and then divide that computed product by the ‘grand total’, that is, ‘expected count = (row total) − (column total)/(grand total)^50^. For these tests, degrees of freedom are used to determine whether a certain null hypothesis can be rejected based on the total number of variables and samples within the experiment. Chi-square analysis is applied to categorical variables.

### The STARNET study

The enrichments of subtype cell clusters associated with sex specificity were examined in 135 tissue-specific GRNs (STARNET.mssm.edu) inferred using block-wise, weighted gene co-expression network analysis and the GENIE3 algorithm applied to genotype and bulk RNA-seq data isolated from aortic arterial wall (AOR), internal mammary artery (MAM), liver, skeletal muscle (SKLM) and subcutaneous (SF) and visceral abdominal fat (VAF) in 582 CAD cases of the STARNET cohort, as described^5,18^. The HighSeq2000 platform was used to sequence RNA to a depth of 25–40 million reads, mostly with the Ribo-Zero protocol. DNA samples were genotyped (*n* = 934/215) with either the OmniExpress Exome array (Illumina, ~900,000 SNPs) or Illumina’s Infinium Global Screening Array 24v1 platform and imputed to a total of 11 million variant calls. Eigengene values were used to establish clinical associations with individual GRNs. GRN contributions to CAD heritability were calculated with the restricted maximum likelihood method, as described^51^. We graded the severity of atherosclerotic disease in STARNET using both the SYNTAX and Duke scores that are established prognostic markers of CAD and are reflective of future clinical outcomes^52,53^.

### Human coronary artery tissue procurement and RNA-seq analysis

Ischemic and nonischemic explanted human coronary artery tissue biospecimens were obtained at Stanford University from diseased heart transplant donors consenting to research studies as described^54^. Hearts were arrested in cardioplegic solution and transported on ice before dissecting proximal coronary artery segments from main branches of left anterior descending, circumflex or right coronary arteries. Epicardial and perivascular adipose was trimmed on ice, rinsed in cold phosphate-buffered saline, rapidly frozen in liquid nitrogen and stored at −80 °C. Normal human coronary artery tissue biospecimens were also obtained at Stanford University from non-diseased donor hearts rejected for orthotopic heart transplantation and processed following the same protocol as hearts for transplant. Tissues were de-identified, and clinical and histopathology information was used to classify ischemic and nonischemic hearts and lesion- and non-lesion-containing arteries. All normal arteries originated from hearts with a left ventricular ejection fraction greater than 50%. Frozen tissues were transferred to the University of Virginia through a material transfer agreement and Institutional Review Board-approved protocols.

Total RNA was extracted from frozen coronary artery segments using the Qiagen miRNeasy Mini RNA Extraction kit (catalog number 217004)^54^. Total RNA libraries were constructed using the Illumina TruSeq Stranded Total RNA Gold kit (catalog number 20020599) and barcoded using Illumina TruSeq RNA unique dual indexes (catalog number 20022371). Individually barcoded libraries passing quality control (QC) were multiplexed and subjected to paired-end 150-bp read sequencing on an Illumina NovaSeq S4 Flowcell (Novogene) to a median depth of 100 million total reads per library. The quality of the reads was assessed using FASTQC, and adapter sequences were trimmed using TrimGalore. Trimmed reads were aligned to the hg38 human reference genome using STAR v2.7.3a. PCR duplicates were marked using Picard, and WASP was used to filter reads prone to mapping bias. Total read counts and reads per kilobase per million mapped reads (RPKM) were calculated with RNA-SeQC v1.1.8 using default parameters and additional flags ‘-n 1000 -noDoC -strictMode’ and GENCODE v30 reference annotation. Transcript and isoform expression levels were estimated using the RSEM package.

### Athero-Express data

Patients eligible to undergo endarterectomy of the carotid or femoral artery to treat atherosclerotic disease in the University Medical Center Utrecht are included in the Athero-Express study. The current number of included AE patients is over 3,500, some 2,400 patients underwent carotid endarterectomy, almost 700 ‘femoral’ patients had surgery, and well over 70 other types of arteries were operated on. All patients are followed for a minimum of 3 years^55^. The Biobank database encompasses many characteristics: pathological specifications, clinical characteristics, 3 years follow-up, whole transcriptome analyses (*n* = 700), whole-genome methylation profile (*n* = 700) and SNP data (*n* = 2,000) and single-cell sequencing data (*n* = 45).

### GRN122, GRN33 and GRN195 validation

#### GRN122, GRN33 and GRN195 conservation

The NetRep R package^31^ was used to assess the preservation of GRN122 and GRN33 using four external independent bulk RNA-seq datasets. GRN122 and GRN33 were accessed by bulk RNA-seq datasets from female patients: two from coronary arteries obtained from (1) 47 freshly explanted hearts from orthotopic heart transplant recipients at Stanford University^30^, (2) 47 postmortem samples downloaded from GTEx (v6)^56^ and one from the aorta, and (3) 70 postmortem samples downloaded from GTEx. The human cell-type-specific RNA-seq dataset was from female patients, 134 primary blood MPs were from STARNET^5^ and 44 primary blood MPs were isolated in the multiethnic NGS-PREDICT study (Ma et al., unpublished manuscript). To assess the preservation of GRN195 using three external independent bulk RNA-seq datasets comprising only males: two from coronary arteries (1) 92 freshly explanted hearts from orthotopic heart transplant recipients at Stanford University and (2) 70 postmortem samples downloaded from GTEx (v6). The third set, 115 HAECs from distinct HAEC cultures generated from the human ECs of male donors^57^.

#### To validate the independent phenotypic association of GRN122, GRN33 and GRN195

The analysis of DEGs was performed on a subset of the Stanford dataset comparing 23 normal against 38 ischemic (*n* = 38) donors as well as 28 lesion-containing coronary arteries against 26 non-lesion-containing coronary arteries. Raw read counts were normalized in DEseq2 (v3.1), and DEGs were called at FDR < 0.05 significance cut-off after correcting for age, sex, RNA integrity number (RIN) score and ancestry. *P* values were adjusted for multiple testing using the Benjamini–Hochberg method.

#### To access the cell specificity of GRN122, GRN33 and GRN195

snATAC-seq was previously performed on 41 coronary artery samples from Stanford using the commercial 10x Genomics Single Cell ATAC platform^30^. In brief, the data were preprocessed using the 10x Genomics Cell Ranger ATAC pipeline (version 1.2.0) and reads mapped to the hg38 reference genome. Downstream snATAC-seq analyses were performed using the ArchR software package (version 1.0.1)^58^ to retain high-quality and informative nuclei with enriched accessibility at transcription start sites. Chromatin accessibility profiles were linked to gene expression by integrating snATAC-seq data with human coronary artery scRNA-seq data from four individuals^23^ to annotate cell types. Briefly, the scATAC-seq gene score (chromatin accessibility) matrix was compared with the scRNA-seq gene expression matrix in ArchR. Each cell in the scATAC-seq space was subsequently assigned the gene expression profile of the closest-matching cell from the coronary scRNA-seq. After integration, the ‘getPeak2GeneLinks’ function in ArchR was used to correlate chromatin accessibility within peaks of integrated RNA expression levels.

### Cell culture and lentiviral transduction

HAECs (TeloHAEC, ATCC CRL-4052, and primary HAECs, ATCC-PCS-100-011) were transduced with lentiviral vectors to overexpress *PLVAP*, *FAM110D* and a non-targeting control (CTL). Cells were maintained in Vascular Cell Basal Medium (ATCC PCS-100-030) supplemented with a VEGF growth kit (ATCC PCS-100-041) and antibiotics. For lentiviral transduction, 75,000 cells ml^−1^ were seeded in a 12-well plate and transduced the next day using an MOI of 2–5 (Supplementary Table 62). Transduced cells were selected with 6 µg ml^−1^ blasticidin (Fisher Scientific) for 6–7 days, after which RNA was extracted using the Monarch Total RNA Miniprep Kit (NEB, catalog number T2010). cDNA was synthesized using the RevertAid First Strand cDNA Synthesis Kit (Thermo Scientific, number K1622), and qPCR was performed using KiCqStart SYBR Green ReadyMix (Sigma-Aldrich, number KCQS02-1250RXN). Gene expression of *PLVAP* and *FAM110D* was quantified using the ΔΔCt method with RPLP0 as the housekeeping gene (Supplementary Table 63).

### RNA-seq

RNA-seq libraries were generated using the QuantSeq 3′ mRNA-Seq V2 Library Prep Kit FWD for Illumina (Lexogen) adhering to the manufacturer’s instructions. For each sample, 100–200 ng of total RNA was used for library preparation with 12 nt unique dual indexes. The resulting libraries were sequenced on the NextSeq 2000 platform (Illumina) using the NextSeq 1000/2000 P2 Reagents (100 cycles) v3 Kit.

### RNA-seq data processing

Sequencing reads were processed using the nf-core RNA-Seq pipeline^59^ (version 3.12.0) with default settings (STAR aligner^60^ and salmon quantification^61^) using the hg38 genome with Ensembl release 98 transcript definitions. Lowly expressed genes were filtered out using the edgeR^62^ (version 3.32.1) function filterByExpr, requiring a minimum of 5 counts in any sample and a minimum total count of 15. Differential gene expression was assessed using DESeq2 (ref. ^63^; version 1.30.1) with default parameters. Gene set enrichment analysis (GSEA) was performed using the fgsea package^64^ (version 1.25.1) using default parameters and the DESeq2 DE statistic as the gene ranking metric. Human Gene Ontology Biological Process gene sets^65^ were obtained from MSigDB^66^ (database release 2023.2.Hs), and gene sets with 10 to 1,000 genes were retained for analysis.

### Proliferation assay

Telo-HAEC cells were cultured in standard growth media, and 7,500 cells per well were seeded in a 96-well plate. To monitor cell proliferation, the plate was placed in the Incucyte S3 Live-Cell Analysis System, with imaging performed every 2 h using the following parameters: ×10 magnification, phase channel and standard scan type. Imaging was carried out for an additional 2–4 days. The incubation time (hours) was determined based on the control wells’ growth curve (focusing on the exponential phase). Data were normalized to 1.0 for all wells by dividing the values at different time points by the value at time point ‘zero’. Figures were generated using GraphPad Prism.

### Cell painting

For cell painting, optically clear flat-bottom 96-well plates (Revvity COL PhenoPlate) were used with a density of 0.6 × 10^4^ HAECs per well. After lentiviral (LV) transduction (MOI 5), selection was performed, and cells were incubated for 6 days. Before fixation with 4% PFA–PBS, mitochondrial staining was performed for 30 min at 37 °C in the dark according to the manufacturer’s instructions in the PhenoVue cell painting JUMP kit (Revvity). After fixation, the cells were washed twice with DPBS followed by incubation for 30 min at room temperature in the dark in a staining solution containing PhenoVue Hoechst 33342 Nuclear Stain, PhenoVue Fluor 512 Nucleic Acid Stain and PhenoVue Fluor 641 Mitochondrial Stain. After washing three times with DPBS, the plates were imaged by using Opera Phenix Plus High Content Screening System (Revvity) with the ×40 water immersion objective and excitation and emission wavelengths as previously recommended^67^. Image analysis was done on maximum-intensity projection images by using Harmony analysis software (Revvity). Cell segmentation allowed the identification of individual cells for measurements. Multiple phenotypic features including morphological, SER texture and Haralick features were extracted by measuring local patterns of intensities from defined regions, including nucleus, ring, cytoplasm, membrane and cell regions (Extended Data Fig. 8). Data obtained from Harmony analysis were exported and ratio normalized relative to the control to assess phenotypic changes^67^.

### Statistics and reproducibility

Carotid endarectomy patients: no statistical method was used to predetermine sample size. No data were excluded from the analysis. The experiments were not blinded or randomized.

*Ldlr*^−/−^*Apob*^100/100^ mice*:* we guarantee to have at least four biological replicates (one biological replicate: one pair of aortic arches) per group to minimize the possible biological variability and the technical background from the Smart-Seq2 technique. As the animals were not subject to any type of treatment, blinding and randomization was not necessary.

Statistical analysis was performed using R (version 4.3.2) and GraphPad Prism9. Data are presented as mean ± s.e.m. using one-way or two-way analysis of variance (ANOVA) with multiple-comparison test or two-tailed *t*-test, or chi-square test, as stated in each figure caption. All graphs were edited for appearance using Adobe Illustrator (v25.2.3).

### Reporting summary

Further information on research design is available in the Nature Portfolio Reporting Summary linked to this article.

## Supplementary information

Supplementary Information Supplementary Fig. 1 Schematic overview of the FACS gating strategy. SSC-A, side-scatter area; FSC-A, forward-scatter area; SSC-H, side-scatter height; CD140B-APC-A, allophycocyanin (APC) anti-human CD140b (PDGFRβ) antibody; CD45-PerCP-A, peridinin–chlorophyll–protein (PerCP) anti-human CD45 antibody; CD31-PE-A, phycoerythrin (PE) anti-human CD31 (PECAM1) antibody; CD144APC-A, APC anti-human CD144 (VE–cadherin) antibody. Forward- and side-scatter measurements estimate the cell size and granularity that are useful for identifying viable single cells. a, Events with low values containing cell debris and red blood cells are excluded by gating based on FCS-A/SSC-A (linear scale). b, Doublet discrimination was implemented using SSC-A/SSC-H. c, Cells were selected for calcein-green AM (Thermo Fisher Scientific) and then sorted for cell-specific antibodies.

Reporting Summary

Supplementary Tables Supplementary Tables 1 and 2: characterisitics of patients with carotid stenosis across genders in the study. Supplementary Tables 3–11: top 100 human-cell-specific gene markers of major cell type markers. Supplementary Tables 12–33: top 100 subcluster-specific markers in carotid atherosclerotic plaque. Supplementary Tables 34–55: top 25 GO processes for subcluster-specific markers in carotid atherosclerotic plaque. Supplementary Tables 56–59: differential expression analysis results for the effect of *FAM110D* and *PLVAP* overexpression in HAECs and Telo-HAECs. Supplementary Tables 60 and 61: Gene Ontology Biological Process enrichment for the effect of *FAM110D* and *PLVAP* overexpression in Telo-HAECs and HAECs under untreated conditions. Supplementary Table 62: transduction with lentiviral vectors to overexpress *PLVAP*, *FAM110D* and a non-targeting control (CTL). Supplementary Table 63: primers to quantify gene expression of *PLVAP* and *FAM110D* with RPLP0 as the housekeeping gene.

## Source data

Source Data Fig. 2 Statistical source data.

Source Data Fig. 3 Statistical source data.

Source Data Fig. 4 Statistical source data.

Source Data Fig. 5 Statistical source data.

Source Data Fig. 6 Statistical source data.

Source Data Fig. 7 Statistical source data.

Source Data Fig. 8 Statistical source data.

Source Data Extended Data Fig. 1 Statistical source data.

Source Data Extended Data Fig. 2 Statistical source data.

Source Data Extended Data Fig. 3 Statistical source data.

Source Data Extended Data Fig. 4 Statistical source data.

Source Data Extended Data Fig. 5 Statistical source data.

Source Data Extended Data Fig. 6 Statistical source data.

Source Data Extended Data Fig. 7 Statistical source data.

Source Data Extended Data Fig. 8 Statistical source data.

## Data Availability

The scRNA-seq data are available on the Gene Expression Omnibus (GEO) database under accession numbers GSE260656 (mouse) and GSE260657 (human). STARNET data are publicly available (controlled access; according to the genomic data access policy and guidelines of NIH) at the dbGaP site (dbGaP study accession: phs001203.v4.p1). Validation data are provided by the HMDP^68^, GTEx^56^ and morbid obesity^69^ studies. Athero-Express Biobank study anonymized data and materials have been made publicly available at DataverseNL and can be accessed at 10.34894/4IKE3T, 10.34894/TYHGEF and 10.34894/D1MDKL and any other data can be provided upon reasonable request from the authors. The scRNA-seq datasets for the coronary and carotid arteries are available on the GEO database under accession numbers GSE131778, GSE155512 and GSE159677 and via Zenodo at 10.5281/zenodo.6032099. All raw and processed single-nucleus chromatin accessibility sequencing datasets are made available on the GEO database under accession numbers GSE175621 and GSE188422. The RNA-seq data related to *PLVAP* and *FAM110D* overexpression have been deposited on the GEO database under accession number GSE287081.

## Code availability

Computer code used for data analysis in the STARNET study is available via GitHub at https://github.com/skoplev/starnet. Code used for data analysis for the scRNA study is available via GitHub at https://github.com/BjorkegrenLab/scRNAseq-HCP.

## References

[1] Bjorkegren, J. L. M. & Lusis, A. J. Atherosclerosis: recent developments. *Cell* **185**, 1630–1645 (2022). 35504280 10.1016/j.cell.2022.04.004 PMC9119695

[2] Rexrode, K. M. et al. The impact of sex and gender on stroke. *Circ. Res.* **130**, 512–528 (2022). 35175851 10.1161/CIRCRESAHA.121.319915 PMC8890686

[3] Hosman, F. L., Engels, S., den Ruijter, H. M. & Exalto, L. G. Call to action for enhanced equity: racial/ethnic diversity and sex differences in stroke symptoms. *Front. Cardiovasc. Med.* **9**, 874239 (2022). 35592405 10.3389/fcvm.2022.874239 PMC9110690

[4] Depuydt, M. A. C. et al. Microanatomy of the human atherosclerotic plaque by single-cell transcriptomics. *Circ. Res.* **127**, 1437–1455 (2020). 32981416 10.1161/CIRCRESAHA.120.316770 PMC7641189

[5] Koplev, S. et al. A mechanistic framework for cardiometabolic and coronary artery diseases. *Nat. Cardiovasc. Res.* **1**, 85–100 (2022). 36276926 10.1038/s44161-021-00009-1 PMC9583458

[6] Ota, H. et al. Sex differences in patients with asymptomatic carotid atherosclerotic plaque: in vivo 3.0-T magnetic resonance study. *Stroke* **41**, 1630–1635 (2010). 20616325 10.1161/STROKEAHA.110.581306

[7] Wendorff, C. et al. Carotid plaque morphology is significantly associated with sex, age, and history of neurological symptoms. *Stroke* **46**, 3213–3219 (2015). 26451032 10.1161/STROKEAHA.115.010558

[8] Mosquera, J. V. et al. Integrative single-cell meta-analysis reveals disease-relevant vascular cell states and markers in human atherosclerosis. *Cell Rep.* **42**, 113380 (2023). 37950869 10.1016/j.celrep.2023.113380 PMC12335892

[9] Burger, F. et al. Single-cell RNA-seq reveals a crosstalk between hyaluronan receptor LYVE-1-expressing macrophages and vascular smooth muscle cells. *Cells* **11**, 411 (2022). 35159221 10.3390/cells11030411 PMC8834524

[10] Alencar, G. F. et al. Stem cell pluripotency genes Klf4 and Oct4 regulate complex SMC phenotypic changes critical in late-stage atherosclerotic lesion pathogenesis. *Circulation* **142**, 2045–2059 (2020). 32674599 10.1161/CIRCULATIONAHA.120.046672 PMC7682794

[11] Zernecke, A. et al. Meta-analysis of leukocyte diversity in atherosclerotic mouse aortas. *Circ. Res.* **127**, 402–426 (2020). 32673538 10.1161/CIRCRESAHA.120.316903 PMC7371244

[12] Pan, H. et al. Single-cell genomics reveals a novel cell state during smooth muscle cell phenotypic switching and potential therapeutic targets for atherosclerosis in mouse and human. *Circulation* **142**, 2060–2075 (2020). 32962412 10.1161/CIRCULATIONAHA.120.048378 PMC8104264

[13] Fernandez, D. M. et al. Single-cell immune landscape of human atherosclerotic plaques. *Nat. Med.* **25**, 1576–1588 (2019). 31591603 10.1038/s41591-019-0590-4 PMC7318784

[14] Winkels, H. et al. Atlas of the immune cell repertoire in mouse atherosclerosis defined by single-cell RNA-sequencing and mass cytometry. *Circ. Res.* **122**, 1675–1688 (2018). 29545366 10.1161/CIRCRESAHA.117.312513 PMC5993603

[15] Gu, W. et al. Adventitial cell atlas of wt (wild type) and ApoE (apolipoprotein E)-deficient mice defined by single-cell RNA sequencing. *Arterioscler. Thromb. Vasc. Biol.* **39**, 1055–1071 (2019). 30943771 10.1161/ATVBAHA.119.312399 PMC6553510

[16] Kim, K. et al. Transcriptome analysis reveals nonfoamy rather than foamy plaque macrophages are proinflammatory in atherosclerotic murine models. *Circ. Res.* **123**, 1127–1142 (2018). 30359200 10.1161/CIRCRESAHA.118.312804 PMC6945121

[17] Cochain, C. et al. Single-cell RNA-seq reveals the transcriptional landscape and heterogeneity of aortic macrophages in murine atherosclerosis. *Circ. Res.* **122**, 1661–1674 (2018). 29545365 10.1161/CIRCRESAHA.117.312509

[18] Franzen, O. et al. Cardiometabolic risk loci share downstream cis- and trans-gene regulation across tissues and diseases. *Science* **353**, 827–830 (2016). 27540175 10.1126/science.aad6970 PMC5534139

[19] Picelli, S. et al. Full-length RNA-seq from single cells using Smart-seq2. *Nat. Protoc.* **9**, 171–181 (2014). 24385147 10.1038/nprot.2014.006

[20] Butler, A., Hoffman, P., Smibert, P., Papalexi, E. & Satija, R. Integrating single-cell transcriptomic data across different conditions, technologies, and species. *Nat. Biotechnol.* **36**, 411–420 (2018). 29608179 10.1038/nbt.4096 PMC6700744

[21] Gomez, D. & Owens, G. K. Smooth muscle cell phenotypic switching in atherosclerosis. *Cardiovasc. Res.* **95**, 156–164 (2012). 22406749 10.1093/cvr/cvs115 PMC3388816

[22] Andueza, A. et al. Endothelial reprogramming by disturbed flow revealed by single-cell RNA and chromatin accessibility study. *Cell Rep.* **33**, 108491 (2020). 33326796 10.1016/j.celrep.2020.108491 PMC7801938

[23] Wirka, R. C. et al. Atheroprotective roles of smooth muscle cell phenotypic modulation and the TCF21 disease gene as revealed by single-cell analysis. *Nat. Med.* **25**, 1280–1289 (2019). 31359001 10.1038/s41591-019-0512-5 PMC7274198

[24] Barabasi, A. L., Gulbahce, N. & Loscalzo, J. Network medicine: a network-based approach to human disease. *Nat. Rev. Genet.* **12**, 56–68 (2011). 21164525 10.1038/nrg2918 PMC3140052

[25] Liu, E., Li, L. & Cheng, L. in *Encyclopedia of Bioinformatics and Computational Biology* (eds Ranganathan, S. et al.) 155–164 (Academic Press, 2019).

[26] Talukdar, H. A. et al. Cross-tissue regulatory gene networks in coronary artery disease. *Cell Syst.* **2**, 196–208 (2016). 27135365 10.1016/j.cels.2016.02.002 PMC4855300

[27] Wang, I. M. et al. Systems analysis of eleven rodent disease models reveals an inflammatome signature and key drivers. *Mol. Syst. Biol.* **8**, 594 (2012). 22806142 10.1038/msb.2012.24 PMC3421440

[28] Diez Benavente, E. et al. Female gene networks are expressed in myofibroblast-like smooth muscle cells in vulnerable atherosclerotic plaques. *Arterioscler. Thromb. Vasc. Biol.* **43**, 1836–1850 (2023). 37589136 10.1161/ATVBAHA.123.319325 PMC10521798

[29] Mocci, G. et al. Single-cell gene-regulatory networks of advanced symptomatic atherosclerosis. *Circ. Res.* **134**, 1405–1423 (2024). 38639096 10.1161/CIRCRESAHA.123.323184 PMC11122742

[30] Turner, A. W. et al. Single-nucleus chromatin accessibility profiling highlights regulatory mechanisms of coronary artery disease risk. *Nat. Genet.* **54**, 804–816 (2022). 35590109 10.1038/s41588-022-01069-0 PMC9203933

[31] Ritchie, S. C. et al. A scalable permutation approach reveals replication and preservation patterns of network modules in large datasets. *Cell Syst.* **3**, 71–82 (2016). 27467248 10.1016/j.cels.2016.06.012

[32] Alsaigh, T., Evans, D., Frankel, D. & Torkamani, A. Decoding the transcriptome of calcified atherosclerotic plaque at single-cell resolution. *Commun. Biol.* **5**, 1084 (2022). 36224302 10.1038/s42003-022-04056-7 PMC9556750

[33] Winkels, H. & Wolf, D. Heterogeneity of T cells in atherosclerosis defined by single-cell RNA-sequencing and cytometry by time of flight. *Arterioscler. Thromb. Vasc. Biol.* **41**, 549–563 (2021). 33267666 10.1161/ATVBAHA.120.312137 PMC7837690

[34] Li, F. et al. Single-cell transcriptional profiling of human carotid plaques reveals a subpopulation of endothelial cells associated with stroke incidences. *J. Cell. Mol. Med.* **26**, 3446–3459 (2022). 35527426 10.1111/jcmm.17354 PMC9189335

[35] Chen, P. Y. et al. Endothelial-to-mesenchymal transition drives atherosclerosis progression. *J. Clin. Invest.* **125**, 4514–4528 (2015). 26517696 10.1172/JCI82719 PMC4665771

[36] Algabri, Y. A., Li, L. & Liu, Z. P. scGENA: a single-cell gene coexpression network analysis framework for clustering cell types and revealing biological mechanisms. *Bioengineering* **9**, 353 (2022). 36004879 10.3390/bioengineering9080353 PMC9405199

[37] Dib, L. et al. Lipid-associated macrophages transition to an inflammatory state in human atherosclerosis increasing the risk of cerebrovascular complications. *Nat. Cardiovasc. Res.* **2**, 656–672 (2023). 38362263 10.1038/s44161-023-00295-x PMC7615632

[38] Islam, S. et al. The mechanobiology of endothelial-to-mesenchymal transition in cardiovascular disease. *Front. Physiol.* **12**, 734215 (2021). 34566697 10.3389/fphys.2021.734215 PMC8458763

[39] Gole, S., Tkachenko, S., Masannat, T., Baylis, R. A. & Cherepanova, O. A. Endothelial-to-mesenchymal transition in atherosclerosis: friend or foe? *Cells* **11**, 2946 (2022). 36230908 10.3390/cells11192946 PMC9563961

[40] Newman, A. A. C. et al. Multiple cell types contribute to the atherosclerotic lesion fibrous cap by PDGFRbeta and bioenergetic mechanisms. *Nat. Metab.* **3**, 166–181 (2021). 33619382 10.1038/s42255-020-00338-8 PMC7905710

[41] Sedding, D. G. et al. Vasa vasorum angiogenesis: key player in the initiation and progression of atherosclerosis and potential target for the treatment of cardiovascular disease. *Front. Immunol.* **9**, 706 (2018). 29719532 10.3389/fimmu.2018.00706 PMC5913371

[42] Virmani, R. et al. Atherosclerotic plaque progression and vulnerability to rupture: angiogenesis as a source of intraplaque hemorrhage. *Arterioscler. Thromb. Vasc. Biol.* **25**, 2054–2061 (2005). 16037567 10.1161/01.ATV.0000178991.71605.18

[43] Xu, J., Lu, X. & Shi, G. P. Vasa vasorum in atherosclerosis and clinical significance. *Int. J. Mol. Sci.* **16**, 11574–11608 (2015). 26006236 10.3390/ijms160511574 PMC4463718

[44] Kolodgie, F. D. et al. Intraplaque hemorrhage and progression of coronary atheroma. *N. Engl. J. Med.* **349**, 2316–2325 (2003). 14668457 10.1056/NEJMoa035655

[45] Skogsberg, J. et al. Transcriptional profiling uncovers a network of cholesterol-responsive atherosclerosis target genes. *PLoS Genet.* **4**, e1000036 (2008). 18369455 10.1371/journal.pgen.1000036 PMC2265530

[46] Kim, D. et al. TopHat2: accurate alignment of transcriptomes in the presence of insertions, deletions and gene fusions. *Genome Biol*. **14**, R36 (2013). 23618408 10.1186/gb-2013-14-4-r36 PMC4053844

[47] Langmead, B. & Salzberg, S. L. Fast gapped-read alignment with Bowtie 2. *Nat. Methods* **9**, 357–359 (2012). 22388286 10.1038/nmeth.1923 PMC3322381

[48] Liao, Y., Smyth, G. K. & Shi, W. featureCounts: an efficient general purpose program for assigning sequence reads to genomic features. *Bioinformatics* **30**, 923–930 (2014). 24227677 10.1093/bioinformatics/btt656

[49] Becht, E. et al. Dimensionality reduction for visualizing single-cell data using UMAP. *Nat. Biotechnol.* **37**, 38–44 (2019). 10.1038/nbt.4314 30531897

[50] Cochran, W. G. Some methods for strengthening the common χ^2^ tests. *Bioethics* **10**, 417–451 (1954).

[51] Zeng, L. et al. Contribution of gene regulatory networks to heritability of coronary artery disease. *J. Am. Coll. Cardiol.* **73**, 2946–2957 (2019). 31196451 10.1016/j.jacc.2019.03.520 PMC6590059

[52] Kovacic, J. C. et al. Comparison of six risk scores in patients with triple vessel coronary artery disease undergoing PCI: competing factors influence mortality, myocardial infarction, and target lesion revascularization. *Catheter. Cardiovasc. Interv.* **82**, 855–868 (2013). 23703934 10.1002/ccd.25008 PMC4155404

[53] Huang, Z. et al. Prognostic value of CAD-RADS classification by coronary CTA in patients with suspected CAD. *BMC Cardiovasc. Disord.* **21**, 476 (2021). 34602055 10.1186/s12872-021-02286-x PMC8487531

[54] Hodonsky, C. J. et al. Multi-ancestry genetic analysis of gene regulation in coronary arteries prioritizes disease risk loci. *Cell Genom.* **4**, 100465 (2023). 38190101 10.1016/j.xgen.2023.100465 PMC10794848

[55] Verhoeven, B. A. et al. Athero-express: differential atherosclerotic plaque expression of mRNA and protein in relation to cardiovascular events and patient characteristics. Rationale and design. *Eur. J. Epidemiol.* **19**, 1127–1133 (2004). 15678794 10.1007/s10564-004-2304-6

[56] Consortium, G. T. Human genomics. The Genotype-Tissue Expression (GTEx) pilot analysis: multitissue gene regulation in humans. *Science* **348**, 648–660 (2015). 25954001 10.1126/science.1262110 PMC4547484

[57] Adelus, M. L. et al. Single cell ‘omic profiles of human aortic endothelial cells in vitro and human atherosclerotic lesions ex vivo reveals heterogeneity of endothelial subtype and response to activating perturbations. *eLife* **12**, RP91729 (2024). 38578680 10.7554/eLife.91729 PMC10997331

[58] Granja, J. M. et al. ArchR is a scalable software package for integrative single-cell chromatin accessibility analysis. *Nat. Genet.* **53**, 403–411 (2021). 33633365 10.1038/s41588-021-00790-6 PMC8012210

[59] Ewels, P. A. et al. The nf-core framework for community-curated bioinformatics pipelines. *Nat. Biotechnol.* **38**, 276–278 (2020). 32055031 10.1038/s41587-020-0439-x

[60] Dobin, A. et al. STAR: ultrafast universal RNA-seq aligner. *Bioinformatics* **29**, 15–21 (2013). 23104886 10.1093/bioinformatics/bts635 PMC3530905

[61] Patro, R., Duggal, G., Love, M. I., Irizarry, R. A. & Kingsford, C. Salmon provides fast and bias-aware quantification of transcript expression. *Nat. Methods* **14**, 417–419 (2017). 28263959 10.1038/nmeth.4197 PMC5600148

[62] Robinson, M. D., McCarthy, D. J. & Smyth, G. K. edgeR: a Bioconductor package for differential expression analysis of digital gene expression data. *Bioinformatics* **26**, 139–140 (2010). 19910308 10.1093/bioinformatics/btp616 PMC2796818

[63] Love, M. I., Huber, W. & Anders, S. Moderated estimation of fold change and dispersion for RNA-seq data with DESeq2. *Genome Biol.* **15**, 550 (2014). 25516281 10.1186/s13059-014-0550-8 PMC4302049

[64] Korotkevich, G. et al. Fast gene set enrichment analysis. Preprint at *bioRxiv*10.1101/060012 (2021).

[65] Gene Ontology Consortium, et al. The Gene Ontology knowledgebase in 2023. *Genetics* **224**, iyad031 (2023). 10.1093/genetics/iyad031 PMC10158837 36866529

[66] Liberzon, A. A description of the Molecular Signatures Database (MSigDB) web site. *Methods Mol. Biol.* **1150**, 153–160 (2014). 24743996 10.1007/978-1-4939-0512-6_9

[67] Cimini, B. A. et al. Optimizing the Cell Painting assay for image-based profiling. *Nat. Protoc.* **18**, 1981–2013 (2023). 37344608 10.1038/s41596-023-00840-9 PMC10536784

[68] Ghazalpour, A. et al. Hybrid mouse diversity panel: a panel of inbred mouse strains suitable for analysis of complex genetic traits. *Mamm. Genome* **23**, 680–692 (2012). 22892838 10.1007/s00335-012-9411-5 PMC3586763

[69] Greenawalt, D. M. et al. A survey of the genetics of stomach, liver, and adipose gene expression from a morbidly obese cohort. *Genome Res.* **21**, 1008–1016 (2011). 21602305 10.1101/gr.112821.110 PMC3129244

[70] Blausen.com Staff. Medical gallery of Blausen Medical 2014. *WikiJournal of Medicine*10.15347/wjm/2014.010 (2014).

