# Supplementary material for: Single-cell RNA sequencing reveals sex differences in the subcellular composition and associated gene-regulatory network activity of human carotid plaques
Source: Nat Cardiovasc Res. 2025 Apr 10;4(4):412–32. doi: 10.1038/s44161-025-00628-y (PMC11994450; doi:10.1038/s44161-025-00628-y)
Supplement: Supplementary file 1 — Supplementary Fig. 1 Schematic overview of the FACS gating strategy. SSC-A, side-scatter area; FSC-A, forward-scatter area; SSC-H, side-scatter height; CD140B-APC-A, allophycocyanin (APC) anti-human CD140b (PDGFRβ) antibody; CD45-PerCP-A, peridinin–chlorophyll–protein (PerCP) anti-human CD45 antibody; CD31-PE-A, phycoerythrin (PE) anti-human CD31 (PECAM1) antibody; CD144APC-A, APC anti-human CD144 (VE–cadherin) antibody. Forward- and side-scatter measurements estimate the cell size and granularity that are useful for identifying viable single cells. a, Events with low values containing cell debris and red blood cells are excluded by gating based on FCS-A/SSC-A (linear scale). b, Doublet discrimination was implemented using SSC-A/SSC-H. c, Cells were selected for calcein-green AM (Thermo Fisher Scientific) and then sorted for cell-specific antibodies. [file 44161_2025_628_MOESM1_ESM.pdf]

nature cardiovascular research

Supplementary information https://doi.org/10.1038/s44161-025-00628-y

Single-cell RNA sequencing reveals sex differences in the subcellular composition and associated gene-regulatory network activity of human carotid plaques

In the format provided by the authors and unedited

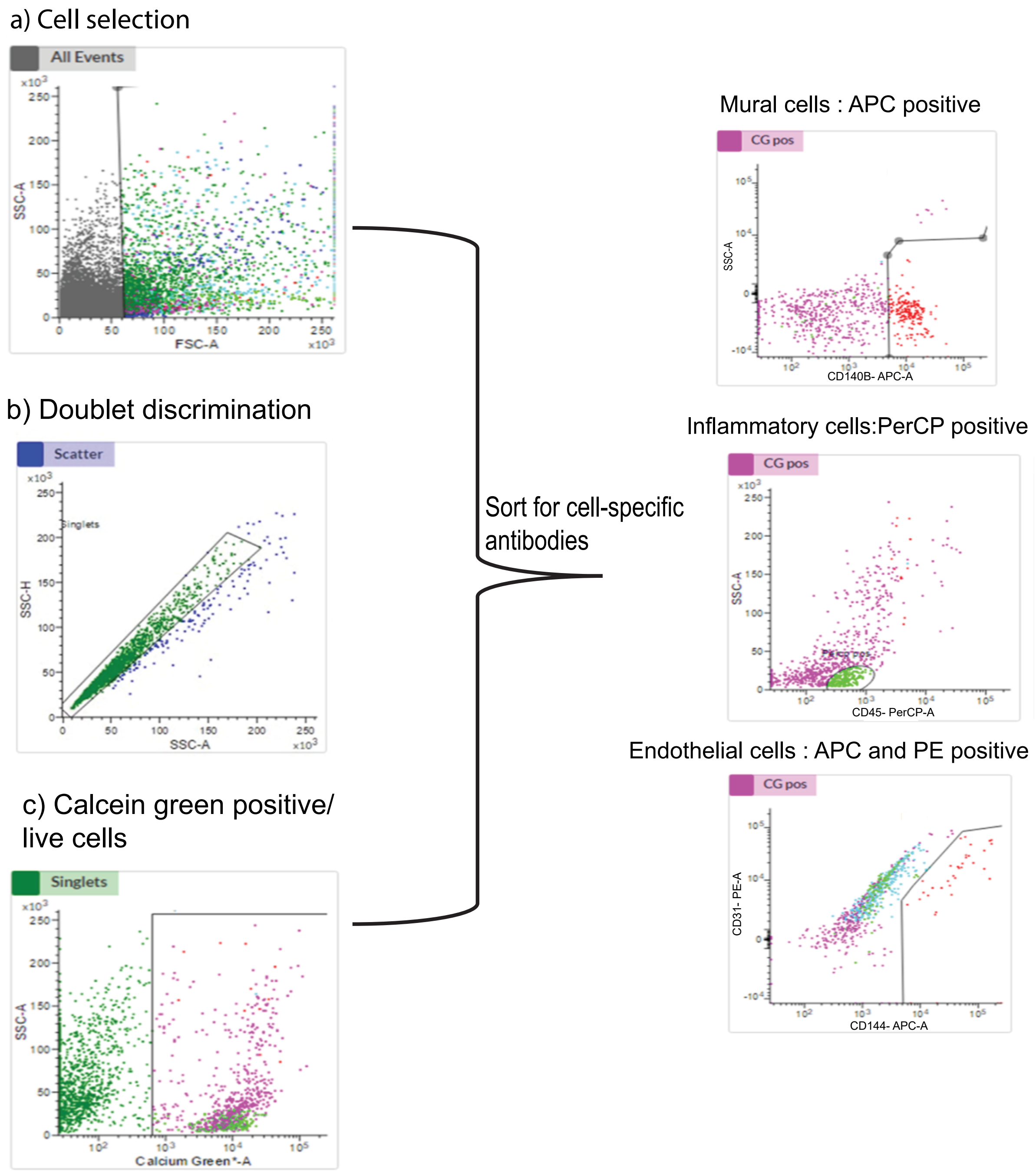
a) Cell selection
All Events
SSC-A
FSC-A
b) Doublet discrimination
Scatter
Singlets
SSC-H
SSC-A
c) Calcein green positive/ live cells
Singlets
SSC-A
Calcium Green*-A
Sort for cell-specific antibodies
Mural cells : APC positive
CG pos
SSC-A
CD140B- APC-A
Inflammatory cells:PerCP positive
CG pos
SSC-A
CD45- PerCP-A
Endothelial cells : APC and PE positive
CG pos
CD31- PE-A
CD144- APC-A
