# Supplementary material for: Single-cell RNA sequencing reveals sex differences in the subcellular composition and associated gene-regulatory network activity of human carotid plaques
Source: Nat Cardiovasc Res. 2025 Apr 10;4(4):412–32. doi: 10.1038/s44161-025-00628-y (PMC11994450; doi:10.1038/s44161-025-00628-y)
Supplement: Supplementary file 2 — Reporting Summary [file 44161_2025_628_MOESM2_ESM.pdf]

nature portfolio

Corresponding author(s): Johan Björkegren, Katyayani Sukhavasi

Last updated by author(s): 22-1-2025

Reporting Summary

Nature Portfolio wishes to improve the reproducibility of the work that we publish. This form provides structure for consistency and transparency in reporting. For further information on Nature Portfolio policies, see our Editorial Policies and the Editorial Policy Checklist.

Statistics

For all statistical analyses, confirm that the following items are present in the figure legend, table legend, main text, or Methods section.

n/a	Confirmed	
☐	☒	The exact sample size (*n*) for each experimental group/condition, given as a discrete number and unit of measurement
☐	☒	A statement on whether measurements were taken from distinct samples or whether the same sample was measured repeatedly
☐	☒	The statistical test(s) used AND whether they are one- or two-sided<br>*Only common tests should be described solely by name; describe more complex techniques in the Methods section.*
☒	☐	A description of all covariates tested
☒	☐	A description of any assumptions or corrections, such as tests of normality and adjustment for multiple comparisons
☐	☒	A full description of the statistical parameters including central tendency (e.g. means) or other basic estimates (e.g. regression coefficient) AND variation (e.g. standard deviation) or associated estimates of uncertainty (e.g. confidence intervals)
☐	☒	For null hypothesis testing, the test statistic (e.g. F, t, r) with confidence intervals, effect sizes, degrees of freedom and P value noted<br>*Give P values as exact values whenever suitable.*
☒	☐	For Bayesian analysis, information on the choice of priors and Markov chain Monte Carlo settings
☒	☐	For hierarchical and complex designs, identification of the appropriate level for tests and full reporting of outcomes
☒	☐	Estimates of effect sizes (e.g. Cohen's *d*, Pearson's *r*), indicating how they were calculated

Our web collection on statistics for biologists contains articles on many of the points above.

Software and code

Policy information about availability of computer code

Data collection	BD FACSChorus™ Software was used for FACS cell sorting by FACS Melody<br>Smart-Seq2 platform was used to generate scRNA seq datasets<br>Sequencing was carried out using HiSeq3000 (Illumina), NextSeq 2000 platform (Illumina), 10x Genomics Single Cell ATAC platform<br>cDNA samples with sufficient quality was analyzed in a 2100 Bioanalyzer (Agilent Biotechnologies)
Data analysis	Statistical analysis tools (e.g., GraphPad Prism version 9.0.2, R version 4.3.2)<br>Bioinformatics software or Data visualization software or packages (e.g., Seurat (5.0.1), Illumina bcl2fastq, TopHat2 with Bowtie2, org.mM.eg.db and org.Hs.eg.db packages (version 3.18.0), Harmony analysis software (Revvity), weighted gene co-expression network analysis (WGCNA) and the GENIE3 algorithm, Network package (1.18.2), NetRep R package, edgeR (version 3.32.1), DESeq2 (version 1.30.1), fgsea package (version 1.25.1), MSigDB (database release 2023.2.Hs), dplyr (1.1.4), ggplot2(3.4.4), Bioconductor, tidyverse (2.0.0), scater, fmsb, umap, tSNE. All graphs were edited for appearance using Adobe Illustrator (v25.2.3)

For manuscripts utilizing custom algorithms or software that are central to the research but not yet described in published literature, software must be made available to editors and reviewers. We strongly encourage code deposition in a community repository (e.g. GitHub). See the Nature Portfolio guidelines for submitting code & software for further information.

Data

Policy information about availability of data

All manuscripts must include a data availability statement. This statement should provide the following information, where applicable:

- Accession codes, unique identifiers, or web links for publicly available datasets
- A description of any restrictions on data availability
- For clinical datasets or third party data, please ensure that the statement adheres to our policy

Single Cell RNAseq data are available at GSE260656 (mouse) and GSE260657 (human).
STARNET data are available at the dbGaP site (dbGaP study accession: phs001203.v3.p1phs001203.v1.p1). Validation data are provided by the HMDP69, GTEx57 and morbid obesity70 studies.
Athero-Express Biobank study anonymized data and materials have been made publicly available at DataverseNL and can be accessed at https://doi.org/10.34894/4IKE3T, https://doi.org/10.34894/TYHGEF, and https://doi.org/10.34894/D1MDKL and any other data can be provided upon reasonable request from the authors.
The single-cell RNA-seq datasets from coronary and carotid artery available at: GSE131778, GSE155512, GSE159677, and zenodo: https://doi.org/10.5281/zenodo.6032099.
All raw and processed single-nucleus chromatin accessibility sequencing datasets are made available on the Gene Expression Omnibus (GEO) database (accessions codes GSE175621 and GSE188422)
The RNA-seq data related to PLVAP and FAM110D overexpression have been deposited in the Gene Expression Omnibus (GEO) under accession number GSE287081

Research involving human participants, their data, or biological material

Policy information about studies with human participants or human data. See also policy information about sex, gender (identity/presentation), and sexual orientation and race, ethnicity and racism.

Reporting on sex and gender

Sex was determined based on self reporting. 15 carefully characterized patients (8 Male and 7 Females) eligible for carotid endarectomy surgery because of significant stenosis (>70%) in the proximal part of their internal carotid arteries were included in the study.

Reporting on race, ethnicity, or other socially relevant groupings

"N/A"

Population characteristics

A: Basic clinic characteristic
Variables Female(n=7) Male (n=8)
mean SD median n mean SD median n
Age (Years) 73.00 7.52 71.00 66.00 9.79 67.00
Symptomatic 3 5
Body Mass Index (kg/m2) 29.68 1.98 29.70 27.70 3.88 27.00

B: Basic blood chemistry
Chol((mmol/l) 4.51 1.21 4.20 4.87 0.32 4.90
LDL (mmol/l) 2.80 0.96 2.53 3.18 0.45 3.37
HDL (mmol/l) 1.35 0.43 1.20 1.10 0.30 1.05
TG 1.76 0.63 1.51 2.23 1.22 1.87

C: Current drug therapies (number of patients)
Statins 7 7
Aspirin 7 7

D: Concomitant diseases (number of patients)
Diabetes Mellitus 2 2
Hypertension 7 5

Recruitment

STARNET has patients who have been diagosed with significant stenosis in internal carotid artery. They can be asymptomatic or symptomatic. Careful clinical and phenotypic information along with blood and metabolic tissues are collected. After consenting to the study, questionnair on risk factors for CAD/atherosclerosis is filled. The study is based on informed consent from patients and hence depends on who is getting operated. In our study females are 4-6 years older than men.

Ethics oversight

Research Ethics Committee of the University of Tartu

Note that full information on the approval of the study protocol must also be provided in the manuscript.

Field-specific reporting

Please select the one below that is the best fit for your research. If you are not sure, read the appropriate sections before making your selection.

☒ Life sciences ☐ Behavioural & social sciences ☐ Ecological, evolutionary & environmental sciences

Life sciences study design

All studies must disclose on these points even when the disclosure is negative.

Sample size	No statistical methods were used to predetermine the sample size. The carotid plaque biopsy is precious invasive biopsy and only patients who are eligible to undergo carotid endarectomy can be involved in this study.
Data exclusions	No data was excluded
Replication	FACS sorting of carotid plaques was performed independently. For mice FACS sorting, 4 biological replicates (one biological replicate: 1 pair of aortic arches) per group to minimize the possible biological variability and the technical background from the SMARTseq2 technique was done
Randomization	Randomization was not done as both human/mice did not require any kind of treatment for this study
Blinding	Blinding was not done as both human/mice did not require any kind of treatment for this study. Informed consent was taken from the patients undergoing carotid endarectomy

Reporting for specific materials, systems and methods

We require information from authors about some types of materials, experimental systems and methods used in many studies. Here, indicate whether each material, system or method listed is relevant to your study. If you are not sure if a list item applies to your research, read the appropriate section before selecting a response.

Materials & experimental systems

n/a	Involved in the study
☐	☒ Antibodies
☐	☒ Eukaryotic cell lines
☒	☐ Palaeontology and archaeology
☐	☒ Animals and other organisms
☐	☒ Clinical data
☒	☐ Dual use research of concern
☒	☐ Plants

Methods

n/a	Involved in the study
☒	☐ ChIP-seq
☐	☒ Flow cytometry
☒	☐ MRI-based neuroimaging

Antibodies

Antibodies used	1. CD31-PE, mouse- (Miltenyi Biotec Cat# 130-102-608, RRID:AB_2657309); Clone ID: 390; 1/200<br>2. CD140b (PDGFRB) - (Thermo Fisher Scientific Cat# 12-1402-81, RRID:AB_529484); Clone ID: Clone APB5, 1/200<br>3. APC/Cyanine7 anti-mouse CD45 - (BioLegend Cat# 103115, RRID:AB_312980); Clone ID : Clone 30-F11; 1/200<br>4. CD31-PE, human -(Miltenyi Biotec Cat# 130-098-173, RRID:AB_2660561); Clone ID: AC128, 1/200<br>5. CD144 (VE-Cadherin) Antibody, anti-human, APC, REAfinity™ - (Miltenyi Biotec Cat# 130-126-010, RRID:AB_2857828); Clone ID: clone REA199; 1/100<br>6. CD140b Antibody, anti-human, APC, REAfinity™ - (Miltenyi Biotec Cat# 130-121-128, RRID:AB_2783953); Clone ID: clone REA363; 1/200<br>7. CD45 Antibody, anti-human, PerCP - (Miltenyi Biotec Cat# 130-113-682, RRID:AB_2726223); Clone ID: clone 5B1, 1/200
Validation	Validations: None available<br>ApplicationS: FC/FACS

Eukaryotic cell lines

Policy information about cell lines and Sex and Gender in Research

Cell line source(s)	Human Aortic Endothelial Cells was purchased directly from ATCC : TeloHAEC, ATCC CRL-4052; and primary HAECs, ATCC-PCS-100-011
Authentication	Human Aortic Endothelial Cells was authenticated by ATCC
Mycoplasma contamination	We routinely test for mycoplasma contamination, the most recent results showing no contamination in any cell lines used.

Commonly misidentified lines (See ICLAC register)	No Commonly misidentified cell lines were used.

Animals and other research organisms

Policy information about studies involving animals; ARRIVE guidelines recommended for reporting animal research, and Sex and Gender in Research

Laboratory animals	Animals (in vivo studies)<br>Species Vendor or Source Background Strain Sex Persistent ID / URL<br>Ldlr-/-Apob100/100 / The JacksonLaboratory/C57Bl6/J; M/F; https://www.jax.org/strain/003000<br><br>The healthy aortic arch in C57Bl6/J mice at baseline (10 weeks) and from atherosclerotic aortic arch in Ldlr-/-Apob100/100 mice (C57Bl6/J) at early (20 and 30 weeks) and advanced (45 and 60 weeks) stages of atherosclerosis progression were used to study the vascular cell transformation. Number of mice used at baseline: 8 mice (2 female, 6 male); early stage: 18 mice (6 female, 12 male) and advanced stage: 20 mice (10 female, 10 male).<br><br>No more than 6 mice were housed in standard, single ventilated cages with 12 h light/dark cycles, ad libitum access to water and fed a chow diet
Wild animals	No wild animals were used in the study
Reporting on sex	Sex was considered in the study
Field-collected samples	No field collected samples were used.
Ethics oversight	Swedish and Finish legislation and local guidelines and regulations for animal welfare and were approved by the Animal Research Ethics committee in Linköping; Sweden (dnr729) and the National Experimental Animal Board of Finland.

Note that full information on the approval of the study protocol must also be provided in the manuscript.

Clinical data

Policy information about clinical studies
All manuscripts should comply with the ICMJE guidelines for publication of clinical research and a completed CONSORT checklist must be included with all submissions.

Clinical trial registration	"N/A"
Study protocol	"N/A"
Data collection	"N/A"
Outcomes	"N/A"

Plants

Seed stocks	"N/A"
Novel plant genotypes	"N/A"
Authentication	"N/A"

Flow Cytometry

Plots

Confirm that:

☒ The axis labels state the marker and fluorochrome used (e.g. CD4-FITC).

☒ The axis scales are clearly visible. Include numbers along axes only for bottom left plot of group (a 'group' is an analysis of identical markers).

☒ All plots are contour plots with outliers or pseudocolor plots.

☒ A numerical value for number of cells or percentage (with statistics) is provided.

Methodology

Sample preparation	the entirety carotid plaques were immediately processed in HBSS solution with 10 mg/ml of Collagenase type I, II and Elastase (Sigma) for 1 hour at room temperature followed by smooth mechanical disruption to obtain a single cell suspension. After centrifugation at 300 × g at 20°c for 5 min, the supernatants were removed and cell pellets resuspended in PBS buffer supplemented with 0.5% bovine serum albumin, 2 mM EDTA, 25 mM HEPES. To enrich for SMCs, ECs and immune cells, the human cell suspensions were labeled with anti-PDGFRβ, anti-CD31/CD144, and anti-CD45 (R&D systems), respectively.
Instrument	FACS Melody
Software	BD FACSChorus™ Software was used
Cell population abundance	The final sorted population was 0.1-4% of the total events
Gating strategy	To maximize yield, we first used a generous gate for forward scatter-area/ side scatter-area (FCS-A/SSC-A, linear scale) only excluding events with low values containing cell debris and red blood cells. Next, doublet discrimination was implemented using FCS-A/FSC-height and SSC-A/SCC-height including a generous threshold for the distance of events from the diagonal line to prevent possible bias toward round shaped cells. Last, cells passing the first two criteria were selected for Calcein-green AM (Thermo-Fisher Scientific) and then sorted for the cell-specific antibodies into individual wells of 384-well plates. To ensure correct gating, samples stained with single, or no antibodies were used as negative controls. During sorting, plates were always kept at 4°C and thereafter immediately stored at −80°C.

☒ Tick this box to confirm that a figure exemplifying the gating strategy is provided in the Supplementary Information.
